# Innovations in graphene-based electrochemical biosensors in healthcare applications

**DOI:** 10.1007/s00604-025-07141-w

**Published:** 2025-04-09

**Authors:** Sudenur Ozbey, Gulsu Keles, Sevinc Kurbanoglu

**Affiliations:** 1 Faculty of Pharmacy, Department of Analytical Chemistry, Ankara University, 06560 Ankara, Türkiye; 2 The Graduate School of Health Sciences, Ankara University, 06110 Ankara, Türkiye

**Keywords:** Graphene, Graphite, Electrochemical sensors, Lab-on-a-chip, Wearable sensors, Point of care diagnosis

## Abstract

The isolation of a single atomic layer of graphite, known as graphene, marked a fundamental moment that transformed the field of materials science. Graphene-based nanomaterials are recognized for their superior biocompatibility compared with many other types of nanomaterials. Moreover, one of the main reasons for the growing interest in graphene is its potential applications in emerging technologies. Its key characteristics, including high electrical conductivity, excellent intrinsic charge carrier mobility, optical transparency, substantial specific surface area, and remarkable mechanical flexibility, position it as an ideal candidate for applications in solar cells and touch screens. Its durability further establishes graphene as a strong contender for developing robust materials. To date, a variety of methods, such as traditional spectroscopic techniques and chromatographic approaches, have been developed for detecting biomolecules, drugs, and heavy metals. Electrochemical methods, known for their portability, selectivity, and impressive sensitivity, offer considerable convenience for both patients and professionals in point-of-care diagnostics. Recent advancements have significantly improved the capacity for rapid and accurate detection of analytes in trace amounts, providing substantial benefits in biosensor technology. Additionally, the integration of nanotechnology has markedly enhanced the sensitivity and selectivity of electrochemical sensors, yielding significantly improved results. Innovations such as point-of-care, lab-on-a-chip, implantable devices, and wearable sensors are discussed in this review.

**Graphical abstract:**

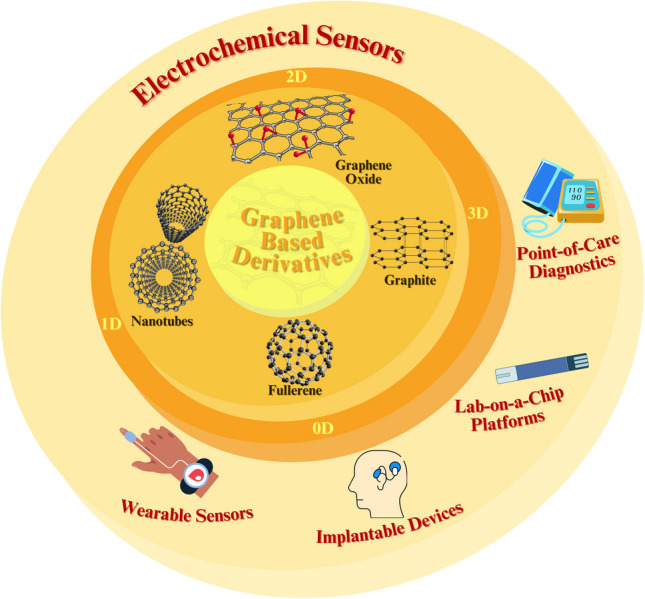

## The origin story of graphene

Long ago, carbon first revealed itself as the essential building block of life, intricately embedded within the fabric of organic and biomolecular structures. As time passed, carbon’s true nature became more apparent, emerging from the depths of the earth in its many guises radiant as diamond, layered as graphite, and elusive as amorphous carbon, paving the way for the journey of discovery that was yet to come [1]. Carbon atoms possess the capacity to form single, double, and triple covalent bonds. Among all the elements, carbon stands uniquely enchanted, capable of weaving long chains of its own kind. Strong, stable carbon–carbon bonds form the basis of an infinite variety of molecular forms. This bonding power allows carbon to create the tiniest structures [2]. This vast array of atomic structures gives rise to a charming spectrum of carbon allotropes, thus to say a “polymorphic parade” each with its own unique physical and functional gifts in various utilizations. Inspired by this diversity, visionary researchers have embarked on pioneering quests to create new nanostructured carbon forms, stretching across dimensions (D) 0D, 1D, 2D, and even 3D, each one promising its own chapter in the evolving story of carbon [3–5]. Then, a new era in material science began, unveiling novel forms of carbon and opening pathways to research and applications since fullerene (C_₆₀_) was discovered by Kroto et al. in 1985 [6].

Another revolutionary discovery, and the very focus of this review, came in 2004 when a single atomic layer of graphite was isolated, graphene (Gr), unveiling a breakthrough that would forever change the landscape of material science [7, 8]. The carbon-based material research won two Nobel Prizes (for graphene and fullerene) and two Kavli Prizes in nanoscience (for carbon nanotubes, to M. Dresselhaus a.k.a. the queen of carbon, and S. Iijima) [9]. Thus, the Nobel Prize in Physics 2010 honors two amazing scientists who have attained the carbon-based materials’ pinnacle: graphene. Andre K. Geim and Konstantin S. Novoselov have conducted the isolation, production, identification, and characterization of graphene. Throughout history, the discovery and advancement of carbon-based materials, including single-walled and multi-walled carbon nanotubes (CNTs), fullerenes, graphite, and graphene, have catalyzed significant scientific and technological breakthroughs. These developments have led to pioneering applications in various fields, including sensor technology, healthcare monitoring, biomedical applications [10], composite materials [11], and microelectronics [12]. Especially the latest sensing applications utilize graphene-based nanomaterials both directly and as substrates for diverse materials [13, 14].

**Fig. 1:**
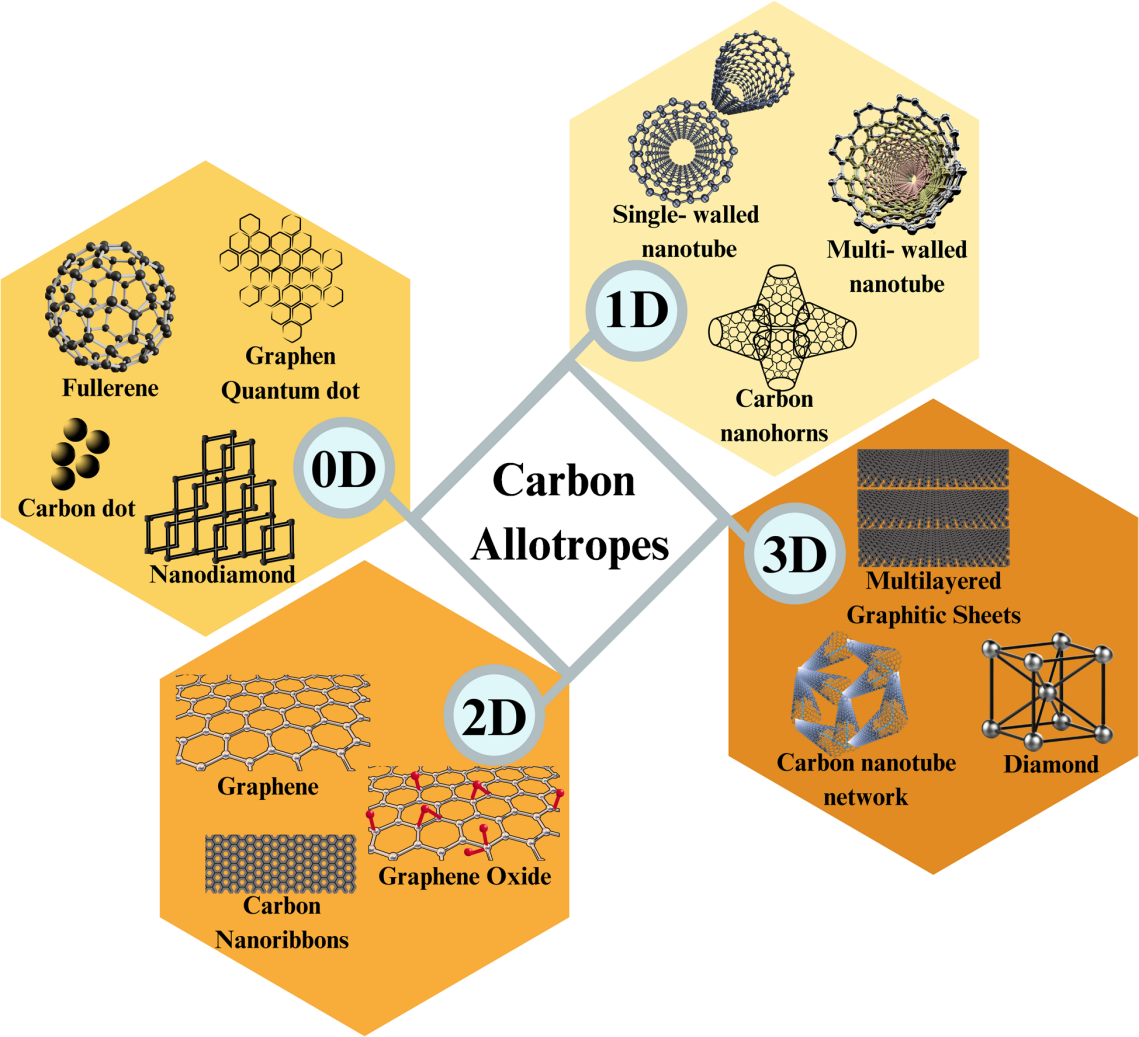
Schematic representation of 0D, 1D, 2D, and 3D carbon-based materials, highlighting their structural differences

In order to examine the origin of graphene, the classification methodology of carbon-based materials must be stated. Carbon-based nanomaterials are derived from their possibility of being in different states of hybridized carbon atoms (sp, sp^2^, and sp^3^) in numerous compounds [15, 16]. This hybridization constitutes the basis of carbon-based nanomaterial classification. This characteristic offers a range of carbon allotrope formations, spanning from individual molecules to layered configurations and intricate mesoporous frameworks. Herein, 0D carbon materials, just as conducting carbon black nanoparticles, nanodiamonds, and the most famous, fullerenes [17, 18], 1-dimensional carbon materials like carbon nanotubes [19, 20], 2D carbon materials such as graphene [21, 22], and 3D carbon materials such as graphite and diamond [1, 23] represent the carbon allotropes. Figure 1 demonstrates these 0D, 1D, 2D, and 3D carbon-based material structure differences. As shown in Fig. 1, 0D materials (e.g., carbon dots, fullerenes) are confined in all directions, while 1D materials (e.g., carbon nanotubes) have elongated structures. 2D materials (e.g., graphene) consist of atomically thin layers, and 3D materials (e.g., carbon nanotube networks) form interconnected porous networks.

**Fig. 2:**
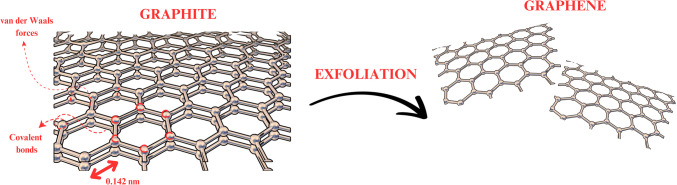
Graphite-to-graphene transformation: exfoliation procedure

To compare by example, on one side stands diamond, known for its exceptional hardness and transparency, which serves as a highly effective electrical insulator while also being a good thermal conductor. Its structure is based on tetrahedral sp^3^ carbon atoms arranged in a unique crystal lattice formed by two interlocking face-centered cubic Bravais lattices [24]. Across from this mighty crystal lies graphite, soft, and shadowed in its opaque form, holding within it a secret, a natural conductor of electricity crafted from delicate stacks of atomic layers. These layers, graphene sheets no thicker than 0.335 nm, are loosely bound by the gentle forces of van der Waals, forming a subtle yet powerful network of sp^2^-hybridized carbon atoms in a hexagonal embrace like a honeycomb [25, 26]. Emerging from the crystalline structure of graphite, the remarkable properties of graphene layers’ exceptional mechanical strength, excellent electrical conductivity, and atomic-scale flexibility have been revealed through advanced scientific inquiry [27]. These monoatomic carbon lattices, arranged in hexagonal perfection, demonstrate fundamental material characteristics that continue to revolutionize modern materials science and nanotechnology applications. Figure 2 illustrates the comprehensive exfoliation process used to convert graphite into graphene layers. It shows the graphite-to-graphene transformation in graphite structure, where van der Waals forces bind the individual graphene layers. When graphite undergoes exfoliation, these forces are disrupted, contributing to the separation and formation of individual graphene sheets [28].

Graphene was the first member of the 2D carbon nanostructure family, and it is described as being more robust than diamond, possessing greater conductivity than copper, and a million times thinner than paper. To visualize, graphene is a single atom thick, and it is tiled in a typical honeycomb lattice with a carbon–carbon distance of 0.142 nm [29]. Graphene’s semi-metallic properties are crucial for exploring its unique physical behaviors, though for materials scientists, the presence of these small band gaps can be undesirable. Numerous strategies have been employed to open the gap, with doping graphene with other materials being the most efficient approach [5]. On the other hand, one of the primary drivers of interest in graphene is its potential for emerging technologies. Its key properties, such as high electrical conductivity [30], high intrinsic charge carrier mobility [27], optical transparency [33], large specific surface area [31], and exceptional mechanical flexibility [32], make it a candidate material for in use in solar cells, touch screens. Additionally, its exceptional durability positions it as a strong contender for the creation of robust materials. Moreover, due to the rapid and efficient movement of charge carriers in graphene, it presents a potential alternative to silicon in high-speed computer chips. From a completely different perspective, graphene has the ability to interact with both RNA and DNA strands, making it a potential candidate for gene delivery applications [33].

Since graphene’s remarkable discovery, the graphene family has continually expanded, unveiling new members with unique structures and properties, each contributing to the evolving landscape of carbon-based materials [10]. The biocompatibility of graphene-based nanomaterials is regarded as superior to that of numerous other types of nanomaterials. This enables graphene composites to function as outstanding foundational elements for the self-organization of functional two-dimensional graphene membranes and three-dimensional graphene/polymer composites [34]. A number of structures derived from graphene have been developed to address certain limitations of graphene and make it more effective, such as cut pieces from a graphene sheet, graphene nanoribbons (GNRs), and nanographenes (NGs) [35]; graphene oxide (GO), reduced graphene oxide (rGO), and graphene quantum dots (GQDs) [36]; and graphene aerogel and graphene hydrogel [37].

### Graphene nanoribbons

Graphene nanoribbons demonstrate a quasi- 1D structure positioned between the 1D structure of carbon nanotubes and the 2D structure of graphene. As previously discussed, significant efforts are being made to engineer a tunable band gap in graphene. Depending on their structure, GNRs, with a bandgap that is inversely related to their width, hold exciting potential because they demonstrate strong gate modulation effects [35]. Among the most promising approaches is the use of GNRs, narrow, flexible strips of graphene with widths on the nanometer scale. Notably, GNRs display a finite band gap when their width is reduced to less than 10 nm, positioning them as compelling candidates for applications in carbon-based nanoelectronics. To obtain GNRs, several advanced techniques were used, including the lithography method (cutting from graphene) [38], chemically oxidizing/longitudinal opened CNTs [39], chemical vapor deposition (CVP) [40], ionic liquid-assisted cleavage of CNTs under microwave irradiation [41], plasma etching [42], lithium intercalation/exfoliation of CNTs [43], and microwave-assisted synthesis [44]. The magnetic and electronic characteristics of GNRs are primarily affected by two key factors: their width and edge structure. Consequently, the precise fabrication of GNRs with atomically precise edges and regulated energy gaps is greatly desired, both for promoting essential physicochemical studies and for their prospective applications in carbon-based nanoelectronics [45]. Unlike armchair-edged GNRs (AGNRs), which exhibit semiconducting behavior with a direct energy gap, zigzag-edged GNRs (ZGNRs) tend to show narrower band gaps and have localized edge states. Moreover, like almost all graphitic-based nanomaterials, GNRs have high electrical conductivity, large surface area, and electrochemical stability, these properties make them logical for use in electrochemical applications [46, 47].

### Graphene quantum dots

Quantum dots have been widely recognized for their exceptional fluorescence-enhancing properties, distinguishing them as a key advancement in the area of nanotechnology. Some well-known kinds of quantum dots include carbon QDs, silver QDs, gold QDs, selenium QDs, and silicon QDs. Following significant theoretical advancements, Ponomarenko and Geim introduced graphene quantum dots in 2008 [48], marking the beginning of their widespread use in biomedical applications. Subsequent research shifted toward GQDs, revealing that they are more appropriate for biological applications compared to other types of quantum dots. In brief, GQDs can be conceptualized as carbon-based, 0D nanomaterials with fluorescent properties composed of a graphene lattice structure. GQDs are exceptionally small, typically measuring less than 20 nm in size, with a reported maximum size of 60 nm, enabling them to simply penetrate biological membranes [49]. Their suspensions exhibit remarkable stability even in environments with high electrolyte concentrations and low pH levels. Moreover, the luminescence properties of GQDs have been thoroughly investigated, underpinning their broad range of biological applications, including biosensing, biological imaging, energy conversion and storage, flexible devices, and catalysis. To fully appreciate the unique characteristics of GQDs, it is useful to compare them with their closest counterparts, carbon dots. GQDs exhibit superior fluorescence [50], a large surface area [51], and high crystallinity [52]. Structurally, GQDs are monolayered, disk-shaped, and composed of sp^2^-hybridized carbon, while carbon dots are primarily spherical with sp^3^ hybridization. The monolayer structure and high crystallinity of GQDs increase their fluorescence, whereas the presence of emitter traps on the surface of carbon dots tends to diminish their luminescence [36].

### Nanographene

As highlighted earlier, nanographene is a tiny graphene fragment, measuring just a few to 10 nm in diameter like the little cousins of regular graphene. These pint-sized forms, also called small GQDs, belong to the exclusive club of aromatic hydrocarbons and are classified as 0D types [53]. Similar to GQDs, NGs can be constructed through diverse techniques. Their finite bandgap enables NGs to be tuned for ultraviolet light absorption and to emit light within the visible spectrum [54]. This distinctive property has garnered significant interest among materials chemists. Consequently, numerous studies have explored the integration of the photoluminescent characteristics of NGs with their mechanical properties, leading to a deeper understanding of their potential applications [55]**.**

### Graphene oxide and reduced graphene oxide

It is not an overstatement to say that graphene oxide and reduced graphene oxide are utilized across a vast sea of literature [56]. The most sophisticated materials developed to overcome the limitations of graphene and to expand its range of applications are GO and rGO. This limitation is primarily influenced by solubility; in contrast to graphene, GO is hydrophilic, which facilitates the preparation of aqueous or organic solvent-based suspensions [57]. Although there are many methods for obtaining GO in the literature, the most common approach is through the chemical oxidation of graphite, which is a multilayer version of graphene, or, more recently, the direct rapid oxidation of graphene. This oxidation is based on the typical 2D graphene structure and results from the transition from sp^2^ to sp^3^ hybridization [58]. In other words, disturbing the crystalline arrangement of graphite creates additional wrinkles and expands the distance between neighboring layers [59]. Subsequently, the graphite oxide multilayers are split apart, resulting in the formation of single-layer GO. Recent research has revealed that GO possesses remarkable properties due to its abundant active oxygen-containing functional groups (= O, –OH, –O–, –COOH). These groups, along with reduced doping elements, serve as catalytic active sites, allowing for covalent or non-covalent modifications tailored to specific application needs. Furthermore, the presence of these oxygen-containing groups expands the interlayer spacing of GO, making it highly hydrophilic and enabling functionalization with small molecules or polymer interlayers [60]. The highly oxidized forms of GO exhibit an electrical bandgap of approximately 2.2 eV, classifying them as electrical insulators. Moreover, GO is often utilized as a precursor for the synthesis of chemically and electrochemically reduced graphene oxide. rGO can be generated by using several techniques, including screen-printing–guided reduction, radiation-induced reduction, solar-mediated reduction, and even multi-step combined techniques. At this stage, rGO represents an excellent compromise between GO. This is due not only to rGO’s graphene-like characteristics, including relatively good electrical conductivity but also to the ease with which it can be produced in the desired quantities from cost-effective GO using various electrochemical methods, as well as microwave and photo-assisted thermal techniques. Notable progress has been made in the functionalization of graphene oxide and reduced graphene oxide, leading to its application in various fields, including drug delivery [61], clean-energy storage devices [62], oil–water separation [63], solar cells [64], desalination [65], catalysis [66], electronic/sensor field [67], and wearable and portable electronics [68].

### Graphene aerogel and graphene hydrogel

Aerogels are sol–gel-derived solid materials with porosity ranging from approximately 80 to 99.8%. Due to its inherent properties, graphene tends to reaggregate with graphite and graphite-like powders because of its strong π–π bonds and Van der Waals interactions in its structure. This can remarkably reduce the specific large surface area, which requires substantial effort to achieve [69]. At this point, researchers have demonstrated that combining the strengths of 3D self-porous aerogels and hydrogels with the properties of graphene can address this issue. These features make graphene aerogels ideal for use as piezoresistive sensing materials due to their compressibility, excellent electrical conductivity, and elasticity [70]. Piezoresistive sensing materials require fast response times, low detection limits, high sensitivity, a wide detection range, and good durability. When used in this capacity, the 3D conductive network of graphene aerogels enhances sensitivity while their compressibility expands the detection range, and their elasticity reduces response time. Typically, graphene aerogels are made by reducing GO and freeze-drying it [71]. However, during the preparation process, GO can easily reaggregate, leading to an unstable and irregular porous structure in the graphene aerogel, which may collapse under significant compressive stress [70]. Additionally, graphene hydrogel fibers hold great potential as electrode materials for next-generation wearable energy storage devices. Consequently, constructing macroscopic monoliths from nanoscale building blocks can notably broaden the applicability of graphene [72, 73].

## Revolutionizing sensors: the graphene effect

Graphene is an excellent conductor of electric charge. Heterogeneous electron transfer (the transfer of electrons between graphene and a molecule in solution, required for oxidation/reduction of the target molecule) occurs at the edges of graphene or defects in its basal plane. Analyzing graphene in more detail has garnered significant attention as a potential matrix for electrochemical biosensors [74]. The incorporation of graphene layers during the biosensor modification process enhances the surface area of the sensor, thereby immobilizing large quantities of antibodies, enzymes, and probe single-stranded DNA (ssDNA) or cells. This, in turn, amplifies the electrochemical detection response, offering heightened sensitivity and performance. Through this method, the biosensor’s ability to detect biological interactions with high specificity is significantly strengthened, positioning graphene as an essential material in the development of next-generation biosensing technologies [75]. Moreover, graphene’s nanoscale dimensions and electronic properties make it an ideal substrate for immobilizing proteins in biochemical sensing. Several properties, such as a wide electrochemical potential range, fast electron transfer rates, and high redox peaks with linear cathodic and anodic currents, have made graphene and its oxidized form (GO) valuable for electrochemical sensors. As mentioned in previous sections, different composite materials have been developed for electrochemical sensing purposes through modifications made to graphene sheets via methods such as electroplating, polymerization, and electrochemical doping [76]. It is also essential to note the contribution of graphene to sensors, which stems from its intriguing physicochemical properties, including its thermal conductivity (5000 W/m·K), exceptional surface area (~ 2630 m^2^/g), and mechanical strength (Young’s modulus of 0.7–1.0 TPa). Due to its high detection capabilities, graphene serves as an excellent molecule for direct electron transfer (DET) reactions when combined with redox enzymes on the electrode surface [77]. Oxygen-containing groups enhance GO’s affinity for aromatic rings and make it highly dispersible in water both essential properties for biosensors. These groups also provide (a) sites for immobilizing biomolecules to detect biological receptors through π–π stacking or hydrogen bonding and (b) GO with fluorescence quenching ability, a critical feature for fluorescence biosensors where energy is transferred from the excited state of the dye to GO [78].

Fluorescence quenching in GO biosensors occurs when fluorophores come into proximity to the sensor. Generally, fluorophores are known to emit photons; however, when situated near GO, they tend to transfer their energy to the GO layer instead [79]. As a result, the fluorescence emission from the dye-labeled probe ceases until it moves away from the GO layer, making this an effective technique for the detection of biomolecules in fluorescence biosensors [80]. In summary, graphene oxide is also a highly promising material for sensors, particularly in applications such as electrochemical, fluorescent, optical, surface plasmon resonance, and surface-enhanced Raman scattering biosensors [81].

## Electrochemical methods in sensing technologies

Electrochemical methods, including voltammetric, impedimetric, and amperometric techniques, have been extensively employed for the detection of an extraordinary array of biological molecules across diverse matrices. These methods assist in measuring different electrical properties, including voltage, current, and charge, along with their correlation to chemical factors, such as concentration, in an electroanalytical system [82]. The most significant advantage these methods offer lies in their potential to create miniaturized analytical devices at micro to nanoscales, enabling the exploration of biological molecules’ effects even on microorganisms and cells within remarkably small operational ranges [83]. On the other hand, potentiometric sensors lie in their simplicity, clear operating principles, precision, and low cost. The application of carbon and metal nanomaterials in potentiometric sensors represents a promising research area, as it enables the development of various nanostructured materials. Recently, literature has begun to highlight POC applications and smartphone-based potentiometric sensors for mobile health. Particularly illuminating are the numerous fields these techniques shed light on, such as the impact of pharmaceuticals on organisms, the effects of pesticides in agricultural settings, and the forensic investigation of pharmaceutical residues. This section will present a concise overview of cyclic voltammetry (CV) and linear sweep voltammetry (LSV), square wave voltammetry (SWV), differential pulse voltammetry (DPV), chronoamperometry (CA), and electrochemical impedance spectroscopy (EIS) techniques [84].

### Cyclic and linear sweep voltammetry

When discussing electrochemical methods, it is essential to begin with “cyclic voltammetry,” the cornerstone of voltammetric techniques. Cyclic voltammetry is based on linear sweep voltammetry and involves the redox active solution’s quantitative measurement of current while the potential is scanned as a function of time [85]. This method evaluates charge changes in electrochemical devices through periodically applied potentials. Here, the slope of the voltage change over time is defined as the scan rate. Cyclic voltammetry has multiple outputs; it elucidates the thermodynamics and kinetics of redox reactions, as well as illuminated chemical mechanisms and enables the adsorption of compounds on the electrode surface. Although cyclic voltammetry and linear sweep voltammetry share similarities, CV does not perform a single scan within a fixed voltage range. Instead, it returns to the starting potential after scanning the designated voltage range, which is why it is termed “cyclic.” The key factor in cyclic voltammetry measurements is the scan rate (*v*). By varying this scan rate and often extending it to linear sweep voltammetry, this technique serves as a versatile tool for deriving mechanistic insights into electrochemical redox reactions and observing reaction intermediates while also assessing product stability [86]. Another essential function of CV is to investigate the redox potential of a molecule or ionic species in the electrolyte by adjusting the electrode potential. The pathway of electron transfer is affected by the interplay between the energy of the electrons at the electrode and the energy states of the redox species [87]. Thus, the direction of the potential scan itself does not dictate whether oxidation or reduction occurs. Furthermore, the observed current provides valuable insights into the reaction rates of the species. The electrode reaction involves several fundamental steps, including mass transfer in the electrolyte, electron transfer at the electrode, and adsorption of species onto the electrode surface [84]. These processes are intricately dependent on the molecular nature of the species, the electrochemical cell, and the measurement conditions. Its applications span a broad spectrum, including medical [88], biological [89], and environmental research [90], establishing it as a shining star in the field [91].

### Differential pulse voltammetry

DPV utilizes small, fixed-size pulses that are layered like a staircase within a linear potential ramp. The fundamental principle of DPV requires measuring the differential current (Δi), which is obtained by subtracting the pre-pulse current from the post-pulse current. This method serves as an ideal approach to minimize non-Faradaic current effects [92]. As a result, voltammograms display peak shapes characterized by an increasing base potential between pulses. Due to the enhanced discrimination of Faradaic currents, DPV surpasses traditional CV [93], facilitating the quantitative detection of analytes even at nanomolar concentrations while exhibiting superior sensitivity and selectivity [94]. Its unique approach significantly reduces charging current interference, thereby elevating detection precision and establishing it as a vital technique in electrochemical analysis [95].

### Square wave voltammetry

It is an electrochemical method characterized by a symmetric square wave superimposed on a staircase potential applied to the working electrode (The magnitude of the pulses is referred to as the square-wave amplitude, denoted as *E*_sw_.), much like in a voltammetric technique [96]. At each step of the staircase ramp, two potential pulses of equal magnitude but opposite directions are applied, forming a single potential cycle in SWV [97]. The length of this potential cycle, which comprises two identical pulses, dictates the frequency of potential cycles per unit time, known as the square wave frequency, *f*. The capacity to reduce background current renders it suitable for obtaining exceptionally low detection limits and for isolating the dynamics of an individual reaction within a mixed solution containing various reacting species [98]. The current response, centered around the redox potential, is determined by comparing forward and reverse currents [99]. Square wave voltammetry also aids in characterizing unknown compounds in solution by determining the electrochemical potential and the number of electrons exchanged in a reaction. The advantages of SWV include exceptional sensitivity, speed, and its ability to discriminate against non-faradaic currents [100]. Moreover, SWV analysis facilitates the investigation of the kinetic and electrochemical mechanisms of analytes, making it invaluable in fields like analytical chemistry, solar energy, thermal energy storage, and the development of molten salt nuclear reactors. It is particularly useful for detecting and characterizing corrosion products and other contaminants in aqueous and molten salt environments [101].

### Chronoamperometry

Amperometric methods are employed to measure the current produced by the analyte under reduction or oxidation conditions at a constant applied potential. Unlike conventional voltammetric techniques, it does not generate a voltammogram, as it maintains a constant potential and deviates from triangular potential sweeps [102]. Typically, in a three-electrode system, a constant voltage is applied between the working and reference electrodes, while the resulting current is measured at the auxiliary electrode. This method records the current over time, and the resulting current is directly proportional to the oxidation or reduction of the analyte, producing a faradaic current [103]. CA excels at analyzing current patterns following significant potential steps, allowing for precise tracking of dynamic electrochemical processes, making the system resistant to potential perturbations. This characteristic provides high selectivity based on the unique oxidation/reduction potentials specific to each analyte [104, 105].

### Electrochemical impedance spectroscopy

EIS is based on applying small-amplitude sinusoidal AC excitation signals and measuring the magnitude and phase of the current passing through the sample at a range of frequencies, producing a complex impedance spectrum. In this method, the fast Fourier transform algorithm, which allows simultaneous analysis, generates multiple frequencies [106]. Through systematic frequency variation, EIS produces impedance spectra, distinguishing the resistive and capacitive components of the circuit from the in-phase and out-of-phase current responses. Nonetheless, this method is extremely susceptible to noise and necessitates advanced, expensive signal processing apparatus. Subsequently, commercial spectrometers generally manage one frequency at a time. Unlike other methods, the expected signal to be measured varies with time or voltage rather than remaining constant with respect to static impedance. Nonetheless, capacitance–voltage measurements still complement impedance spectroscopy by providing valuable insights into the electrical characteristics of the sample under investigation. EIS has been employed in material characterization for various reasons, particularly because it enables differentiation between interface phenomena and bulk properties. It finds broad applications across fields such as energy conversion [107], electrocatalysis [108], corrosion studies [109], chemical sensing, and non-invasive diagnostics [110]. Furthermore, since the electrical features of materials are highly dependent on their interactions with the environment, EIS proves valuable in sensing and biosensing applications [111].

## Overview of electrochemical biosensor innovations

Sensors are instruments that detect and convert environmental or biological signals into measurable electrical pulses and have revolutionized a wide range of scientific and technical fields. In particular, electrochemical sensing devices use electrochemical reactions to detect the presence or concentration of chemical substances. Such sensors rely on reduction and oxidation (redox) reactions, which occur simultaneously between the analyte in a solution and electrodes to convert chemical information into an interpretable electrical signal [112]. An electrochemical sensor functions by enabling a chemical reaction at the working electrode, where the target analyte engages with the electrode surface. This interaction leads to the generation of an electrical current, voltage, or impedance, which can be associated with the concentration of the analyzed material. The performance of these sensors is contingent upon the material and design of the electrodes, as well as any modifiers that may be applied to optimize selectivity and sensitivity. Hence, they are essential in numerous contexts, from biomedical devices to environmental pollution analysis [113].

Electrochemical sensors are classified into three principal categories, namely, potentiometric, amperometric, and voltammetric. Potentiometric sensors are designed to measure potential differences between electrodes and reference electrodes, whereas amperometric sensors are utilized to quantify currents between electrodes. In a voltammetric sensor configuration, the analyte concentration is determined through a measurement process involving the variation of voltage, resulting in the generation of a current signal that carries information about the analyte concentration [114]. These sensors are frequently employed to discern the reduction or oxidation potential of a chemical reaction. The composition of the working electrode and the modification applied to its surface are of great significance in optimizing the performance of a sensor. The utilization of conventional electrode materials has witnessed a gradual substitution with carbon-derived alternatives comprising glassy carbon electrodes (GCEs), carbon paste electrodes (CPEs), screen-printed carbon electrodes (SPCEs), and laser-induced carbon electrodes (LICEs). These carbon-based materials exhibit enhanced electrochemical properties, including elevated conductivity, augmented surface area, and greater sensitivity, thereby rendering them more efficacious and discerning for contemporary applications in modern sensor applications [115].

Additionally, the incorporation of nanotechnology has led to a notable enhancement in the sensitivity and selectivity of electrochemical sensors with markedly elevated findings. A wide variety of nanomaterials is engaged in research in this respect, such as nanoparticles, quantum dots, fullerenes, and graphene, which are deposited on electrode surfaces, thereby increasing the surface area and catalytic activity and, thus diminishing detection limits [116]. Gold nanoparticles, for instance, act as catalytic agents at the electrode interface, enhancing the rate of reactions, whereas carbon nanotubes contribute by offering superior electrical conductivity and configurational stability. This synergy promotes more effective electron transfer, subsequently ensuring enhanced overall performance of the electrochemical sensor [117].

One might consider electrochemical sensors to be skilled interpreters, each fluent in a different “language” of nature, whether it be the language of light, pressure, chemistry, or biology. Like a keen-eyed observer, an optical sensor “sees” and translates changes in light intensity, while a thermal sensor “feels” shifts in temperature like a thermometer in a human hand. Chemical sensors “sniff out” the presence of gases or shifts in pH, much like a finely tuned nose identifying subtle scents carried through the air [118]. Biosensors act as biological detectives, using enzymes or antibodies to recognize specific molecules, much like a puzzle piece seamlessly finding its perfect match. Magnetic sensors, in turn, “sense” invisible magnetic fields, translating them into measurable signals, much like a compass navigating the unseen forces of the earth. Each sensor uniquely decodes its environment, translating subtle changes into information we can interpret and use [119].

**Fig. 3:**
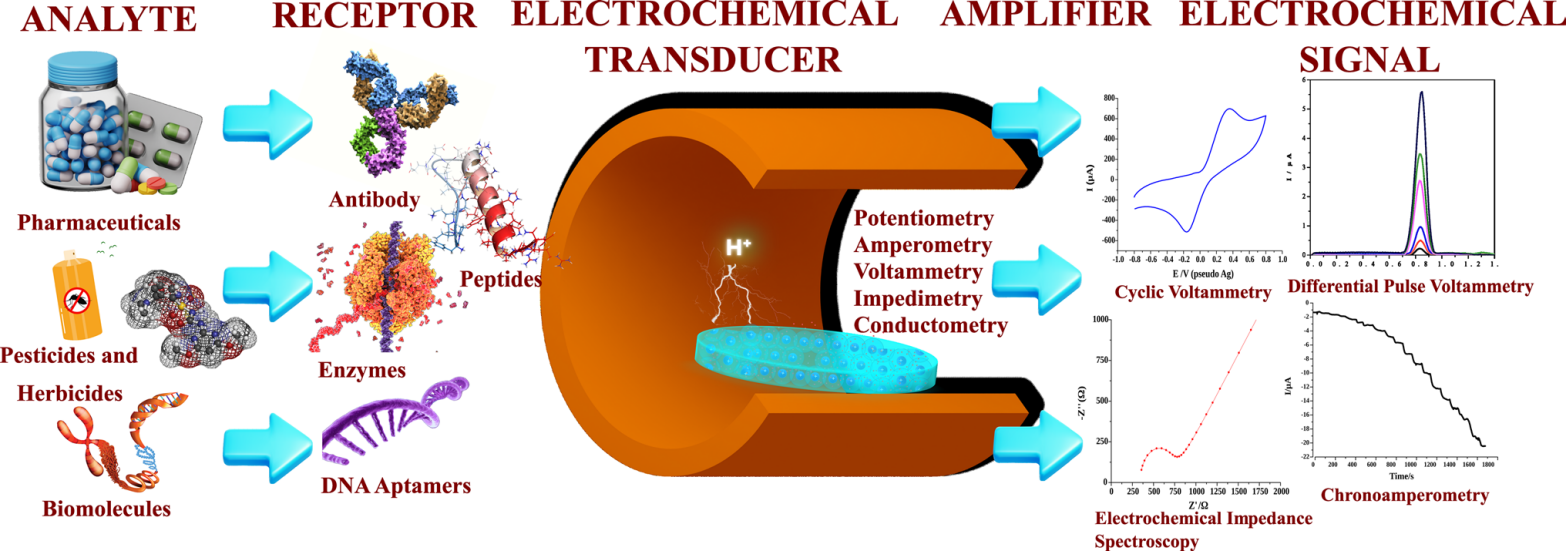
Illustration of the working principle of electrochemical biosensors

Being a variety of electrochemical sensors, biosensors are instruments that integrate the intricate functionality of biological systems with sophisticated technological and engineering capabilities, enabling the assessment of biomolecular interactions and biological processes. In essence, biosensor functions by initiating a chemical or physical interaction with a specific biological target molecule—such as an enzyme, antibody, nucleic acid, or cell—which is then quantified through the conversion of the interaction into a meaningful electrical signal as shown in Fig. 3 [120]. Biosensors operate much like a lock and key mechanism, where the biological recognition elements act as the “lock” that is finely tuned to detect a specific “key”—the target analyte. Once the analyte fits into this biological lock, a physicochemical transducer works as the messenger, transforming this molecular interaction into a measurable signal, resembling a translator converting one language to another. This seamless collaboration between the biological detector and the transducer enables the biosensor to effectively detect and quantify specific compounds within a sample [121].

**Fig. 4:**
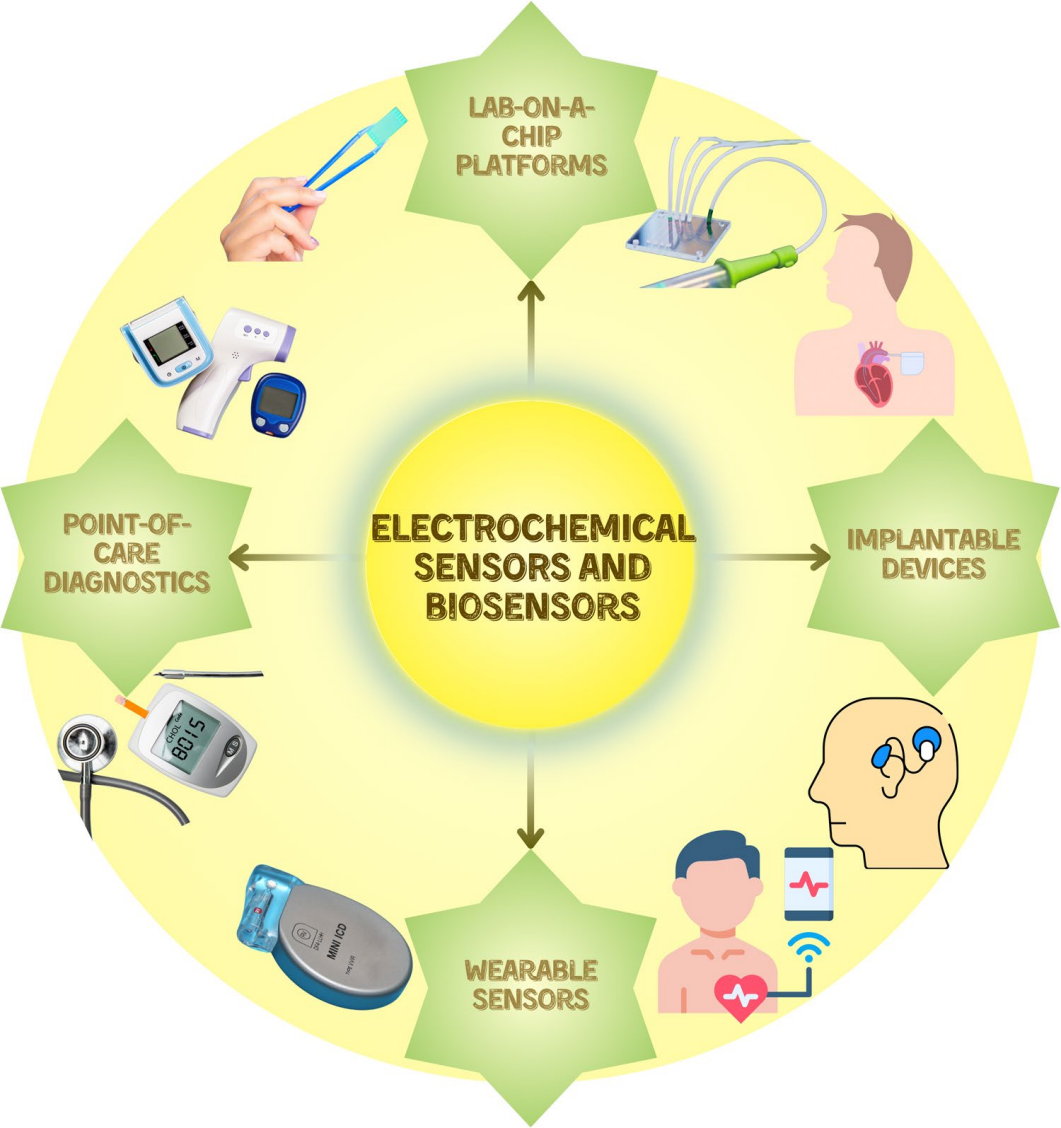
Applications of electrochemical biosensors in medicinal diagnostics

In addition, the potential for the miniaturization of biosensors has paved the way for the development of portable, hand-held devices capable of non-invasive applications. This qualifies them as an excellent choice for personal health monitoring, where the use of large laboratory equipment is not feasible. The affordability, high sensitivity, and extensive range of potential applications offered by this technology suggest that biosensors will become even more prevalent in the future [122]. Even more remarkably, the potential applications of electrochemical sensor technology have extended well beyond the medical field as research is being conducted in a considerable number of other areas, including environmental monitoring, wastewater analysis, and food safety. To illustrate, chemical sensors are crucial devices in the fight against environmental contamination, as they are capable of detecting the presence of heavy metal ions in aqueous environments. It can thus be concluded that electrochemical biosensors have been positioned as fundamental instruments, also, as illustrated in Fig. 4, in the fields of point-of-care (POC), lab-on-a-chip (LOC), wearable, and implantable devices [123].

## Applications of graphene-based electrochemical biosensors in healthcare

As previously discussed, electrochemical biosensors are playing a vital role in various fields, especially in medical diagnostics, offering rapid, sensitive, and cost-effective analysis. These sensors are capable of detecting biomolecules through electrochemical reactions, which are then transformed into electrical signals that can be quantified [124]. In a nutshell, these sensors facilitate the precise detection of particular biomarkers, such as proteins, enzymes, or nucleic acids, which are indicators of disorders, including cancer, heart-related ailments, and infections. For instance, sensors designed to recognize prostate-specific antigen (PSA) offer a powerful tool for the early identification of prostate cancer [125]. In a similar manner, electrochemical DNA sensors can be engaged to detect genetic alterations or pinpoint viral infections, enabling precise diagnostic capabilities. Besides, electrochemical sensors represent a reliable, real-time method for monitoring electrolyte concentrations, including sodium, potassium, and chloride. These electrolytes serve as critical indicators of health conditions associated with kidney disorders, dehydration, and heart disease [126].

Herein, it is of significant importance to acknowledge glucose sensors since they are essential tools for diabetics, as they employ electrochemical principles based on glucose oxidation to monitor blood glucose levels. They may be implanted, attached to the body by means of a wearable device, or administered via microneedles, allowing the clinician to select the most comfortable method for each patient. As a result of recent advancements, the ability to rapidly and accurately detect analytes in trace quantities has been significantly improved, furnishing immense advantages in biosensor technology [127].

### Wearable sensors

Wearable sensors are cutting-edge technologies embedded in clothing and accessories or worn directly on the body to monitor an array of physiological and environmental parameters. They are the gold standard for continuous, real-time health tracking and are engaged extensively in healthcare, fitness, and wellness applications [128]. These monitoring devices monitor key health metrics, such as heart rate, body temperature, blood pressure, and oxygen levels, making them particularly beneficial for managing long-term health issues, just as glucose monitors track blood sugar levels continuously without needing frequent blood samples [129].

A further instance, sweat-based wearable biosensors stand out as pertinent and may regarded as one of the most remarkable examples of electrochemical biosensors. The inspection of metabolites and electrolytes in perspiration enables the real-time provision of patients’ health status. As a case in point, biosensors that quantify lactate levels in sweat could be used to monitor athletes’ performance and muscle fatigue [130]. Perspiration-sensing wearables are constructed from flexible and lightweight materials, which are affixed to the dermal tissue and are designed to be used over an extended period of time. Since lactate levels function as an indicator of muscle metabolism, these sensors are especially useful for evaluating performance during training [131].

Adopting a distinct approach, Phamonpon et al. proposed an innovative bioelectrode platform for electrochemical monitoring of sweat lactate in a very recent study, utilizing platinum nanoparticles (PtNPs) combined with rGO to modify carbonized silk cocoon. As the biorecognition element, lactate oxidase (LOx) was immobilized to the array. The subsequent utilization of scanning electron microscopy (SEM), atomic force microscopy (AFM), and X-ray photoelectron spectroscopy (XPS) have validated that PtNPs supported on rGO were effectively deposited onto the surfaces of carbonized silk cocoons, which were characterized by their physical and chemical modifications. The electrocatalytic performance of PtNPs and the elevated surface area, alongside the functional properties of rGO, markedly enhanced the electrochemical reactivity of the sensor in the recognition of lactate. The novel bioelectrode array exhibited a selective capacity to identify sweat lactate concentrations within the spectrum of 0–25 mM, demonstrating a limit of detection (LOD) of 0.07 mM. This sensitivity is adequate for differentiating between individuals with normal physiological conditions and those predisposed to muscle fatigue, utilizing a threshold sweat lactate level of 12.5 mM. The patch testing conducted on a cohort of 20 healthy volunteers (comprising 10 males and 10 females) assessed the dermal allergic and irritative responses elicited by PtNPs/rGO-carbonized silk bioelectrode. The findings demonstrated that the bioelectrode did not induce any irritant or allergic contact dermatitis, thus affirming the safety profile of its chemical constituents for the human epidermis. This implies a substantial potential for its utilization as a wearable lactate sensor in direct epidermal contact with human skin [132].

Owing to their metallic properties and excellent electrical conductivity (2400 S/cm), Ti_3_C_2_T_x_ MXene nanosheets hold promise for electrochemical applications, yet their use in wearable and flexible biomarker detection in sweat remains largely unexplored [133]. In this respect, Nah et al. introduced an impedimetric immunosensor integrating microfluidics utilizing Ti_3_C_2_T_x_ MXene laser-burned graphene (LBG) to monitor sweat cortical in a non-invasive manner. The wearable device was fabricated with polydimethylsiloxane (PDMS) as a substrate, onto which LBG was transferred by removing the polyamide film. To address the issue of inter-flake disconnection caused by laser burning, Ti_3_C_2_T_x_ MXene was added to the electrode to harness its enhanced conductivity. The biosensor was completed by attaching a cortisol antibody to the working electrode. Subsequent electrochemical calibration with the EIS method revealed the sensor’s superior capacity in detecting sweat cortisol as a wearable healthcare device with a dynamic range from 0.01 to 100 nM and a LOD of 1 pg/mL. The selectivity of the Ti_3_C_2_T_x_ MXene/LBG cortisol biosensor was evaluated by testing its response to interference from other steroid hormones in artificial sweat, showing minimal changes in *R*_ct_ compared to cortisol. Additionally, reproducibility was assessed by testing eight sensors with a low relative standard deviation (RSD) of 4.6%, indicating reliable performance for cortisol detection. The wearable cortisol patch was employed to detect cortisol in sweat collected during exercise for real-sample analysis, with spiked cortisol biomarkers assessed using the standard addition method. The device demonstrated a 2.80% RSD across four samples, highlighting its potential for point-of-care (POC) cortisol biomarker monitoring [134].

**Fig. 5:**
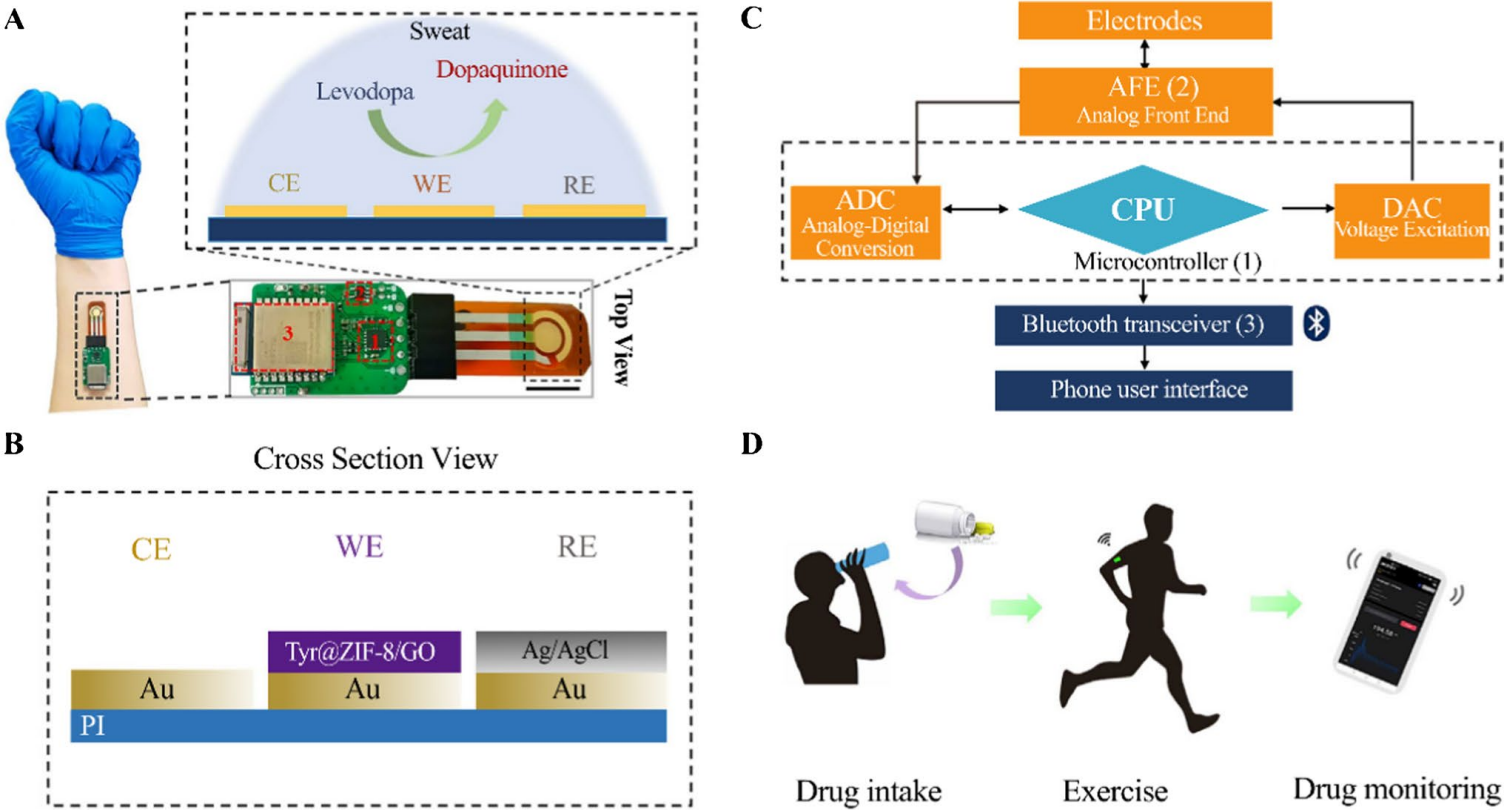
Schematic diagram of the wearable levodopa sweat biosensor. Reprinted with permission from [137]

Levodopa (l-dopa) is a frequently employed treatment for Parkinson’s disease; nevertheless, excessive intake, whether due to overdose or consumption of natural dietary sources, can pose significant health risks, including tardive dyskinesia [135]. Thus, continuous monitoring of l-dopa levels in the body is imperative for individuals undergoing treatment to ensure safe and effective management of dosing [136]. Regarding this, Xiao et al. designed a noninvasive, wearable, and portable biosensor to track alterations of l-dopa in the human body. As shown in Fig. 5, the construction process involves integrating metal–organic frameworks (MOFs) with an enzyme-functionalized electrochemical sensor array to monitor the target analyte in sweat. This is achieved through the in-situ growth of Zeolitic Imidazolate Framework- 8 (ZIF- 8) nanoparticles on GO, followed by the immobilization of tyrosinase onto the resulting ZIF- 8/GO composite. The fusion of the wireless electronic module facilitated uninterrupted communication between the sensor and the smartphone application, enabling continuous analysis. Applying the CA technique, the sensor revealed a LOD of 0.45 µM with a wide dynamic range from 1 to 95 µM. Further investigations were conducted on volunteers who consumed 500 g of broad beans to assess the feasibility of the newly developed sensor, as broad beans are a natural source of levodopa. To minimize dietary interference, participants fasted for 12 h before the test. Sweat accumulation was stimulated through stationary cycling, allowing continuous monitoring of levodopa levels, which peaked at approximately 4 µM after 45 min. The sensor’s reliability was confirmed by comparing wireless device readings with electrochemical workstation measurements, demonstrating a strong correlation (Pearson coefficient: 0.9867). It can thus be concluded that the novel design and enzyme immobilization method holds considerable potential for non-invasive monitoring of various target analytes through wearable biosensors [137].

The ketogenic diet has attracted significant interest due to its ability to effectively treat chronic diseases. Nevertheless, adherence to such restrictive dietary regimens over an extended period can result in severe health complications. Despite the significant progress made in diagnostic methodologies and therapeutic approaches within contemporary medicine, there persists a considerable deficiency in the personalized health management necessitated by this nutritional strategy [138, 139]. Thus, Wang et al. proposed a minimally invasive, wearable biosensor to monitor ketone and glucose levels. The microneedle array demonstrates superior mechanical characteristics, facilitating the reliable collection of interstitial biomarkers while simultaneously mitigating the discomfort commonly linked to dermal puncture. The fabrication of biosensors involved cleaning ceramic substrates and growing graphene nanowalls through plasma-enhanced chemical vapor deposition (CVD), followed by modification with chitosan, TBO, carbon nanotubes, and enzymes such as *β*-hydroxybutyrate dehydrogenase (HBD) and glucose oxidase (GOx). The electrodes were prepared by drop-casting enzyme solutions onto the surfaces, allowing them to dry, and were stored at 4 °C overnight for further use in HB and glucose sensing. Integrating vertical graphene yielded remarkable electrical conductivity, achieving a sensing platform characterized by substantial sensitivity (234.18 μA mM^−1^ cm^−2^) and a notable detection threshold of 1.21 μM. Upon evaluation with human participants, the fully integrated biosensor proficiently monitored dynamic metabolite concentrations and demonstrated a satisfactory correlation with standardized blood assessment methodologies. Hence, one can claim that this viable and effective sensing platform possesses the capacity to facilitate the implementation of ketogenic diets in the realms of personal nutrition and wellness optimization [140]. In addition to these discussions, recent studies about graphene-based wearable sensor platforms are tabulated in Table 1.

**Table 1:** Some selected studies on graphene-based wearable sensor platforms and their applications

**Analyte**	**Electrode**	**Method**	**Linear Range**	**LOD**	**Application**	**REF**
H_2_O_2_	PI/LIG/BPE	ECL	1–100 µM	5.8729 µM	Human serum	[141]
Glucose	GOx/PI/LIG/BPE		1–100 µM	0.138 µM		
Cortisol	Cortisol Abs/Ti_3_C_2_T_x_ MXene/LBG/PDMS	EIS	0.01–100 nM	1 pg/mL	Sweat	[134]
H_2_O_2_	PB-rGO/FTO	AMP	0–6 mM	1.6 μM	Sweat beverages	[142]
Glucose	GOx/PB-rGO/FTO	CA	0–2.1 mM	7.94 μM		
Levodopa	Tyrosinase/MOF/ZIF- 8/GO/AuE	CA	1–95 µM	0.45 µM	Artificial sweat	[137]
Glucose	GOx-gel-rGO-Au/SPGE	CA	1.25–850 μM 0.85–7.72 mM	1.25 μM	Human sweat	[143]
*β*-hydroxybutyrate	HBD/NAD^+^/TBO-CNTs-CS/VG	CA	0.1–1.0 mM	8.51 μM	Human ISF	[140]
Glucose	GOx/TBO-CNTs-CS/VG		1–8 mM	1.21 μM		
Sweat Lactate	LOx/CeO_2_/MoS_2_/AuNPs/LSGE	CA	0.1–50 mM	0.135 mM	Artificial sweat	[144]
Methyl Parathion	Lipase/LIG-AuNPs/PI film/PDMS	DPV	0.001 − 200 μM	0.646 nM	Epipremnum aureum Lettuce leaves	[145]
Lactate	LOx/PtNPs/rGO-carbonized silk	CA	0–25 mM	0.07 mM	Human sweat	[132]

Abbreviations: *Ab*: antibody, *AMP*: amperometry, *AuE*: gold electrode, *AuNP*: gold nanoparticles, *BPE*: bipolar electrode, *ECL*: electrochemiluminescence, *FTO*: fluorine- doped tin oxide, *H*_2_*O*_2_: hydrogen peroxide, *ISF*:interstitial fluid, *LIG*: laser-induced graphene, *LSGE*: laser-scribed graphene electrode, *MoS*_2_: molybdenum disulfide, *NAD*^+^: nicotinamide adenine dinucleotide, *PB*: Prussian blue, *PI*: polyimide, *SPGE*: screen-printed graphene electrode, *TBO*: toluidine blue O, *VG*: vertical graphene

### Lab-on-a-chip platforms

The advent of personalized medicine has effectively removed the constraints previously imposed on individuals seeking health evaluations, which were often lengthy and required visits to healthcare facilities. Conversely, there is an emerging preference for the use of portable diagnostic instruments intended for individual utilization, offering convenience and autonomy in the realms of health monitoring and assessment. In this respect, lab-on-a-chip (LOC) platforms are exceptionally compact devices that consolidate various laboratory functions onto a single, small-scale chip, usually just a few millimeters or centimeters in dimension [146, 147]. These systems are engineered to perform multiple analytical processes, such as sample handling, chemical reactions, separations, and detections, within a unified, miniaturized setup. Through the integration of traditionally separate laboratory steps into one streamlined unit, LOC devices achieve the fast and efficient real-time analysis of chemical or biological specimens, enhancing diagnostic and analytical capabilities across numerous fields [148].

LOC technology has been incorporated into health diagnostics by means of biomarker detection or genetic material analysis, including SARS-CoV- 2 diagnostic assay, as well as in pharmaceutical development studies since these platforms are capable of detecting multiple drug candidates concurrently [149]. Besides, they are not only practical in environmental monitoring but also in forensic investigations resulting from the integration of reduced sample and reagent use, along with automation and portability, achieving rapid DNA tracing with susceptible fingerprint recognition. These attributes significantly enhance the efficiency of laboratory processes by minimizing waste and human intervention while also speeding up analysis and improving accuracy [150].

Serum PSA constitutes a significant biomarker that is extensively employed in the early identification and surveillance of prostate carcinoma. This glycoprotein, primarily synthesized by the epithelial cells of the prostate gland, acts as a pivotal indicator of prostatic health. Its increased concentrations within the circulatory system may signify the existence of prostate cancer, thereby underscoring the vital importance of early detection [151, 152]. Hence, Dou et al. introduced an innovative electrochemical immunochromatography (EIC) approach consisting of two distinct parts. The first part comprises a biosensor chip integrating an SPCE, which was modified with gold nanoflowers (AuNFs). The second part had an integrated circuit (IC) architecture situated on the superior layer of the biosensor chip. The AuNFs exhibiting a superior specific surface area were electrodeposited onto the SPCE interface, thereby offering an increased number of adsorption sites for the PSA capture antibody (cAb) and facilitating the efficient recognition of PSA. The investigation refined the performance of the PSA EIC biosensor by systematically assessing the concentration of the PSA cAb (ranging from 10 to 200 µg/mL) alongside the duration of the chromatographic reaction, between 6 and 20 min. The optimal signal and effective immobilization were attained with an antibody concentration of 100 µg/mL. A reaction time of 15 min yielded the most favorable signal-to-noise ratio, whereas extending the duration to 20 min resulted in a diminished signal attributable to excessive adsorption of PSA mAb–horseradish peroxidase, thereby establishing 15 min as the superior parameter for subsequent investigations. As a result, the research achieved a satisfactory linear range extending from 0 to 100 ng/mL, while the LOD was determined to be 8.78 fM, with an overall reaction duration of under 20 min. Furthermore, in addition to exhibiting femtomolar sensitivity, the biosensor chips demonstrated selective recognition of prostate cancer biomarkers present in serum samples. Through the identification of the PSA biomarker in clinical serum samples, the EIC biosensor chip effectively differentiated prostate cancer patients from healthy control subjects (*p* < 0.001). Furthermore, this EIC system was user-friendly, requiring neither specialized expertise nor demanding instrumentation [153].

**Fig. 6:**
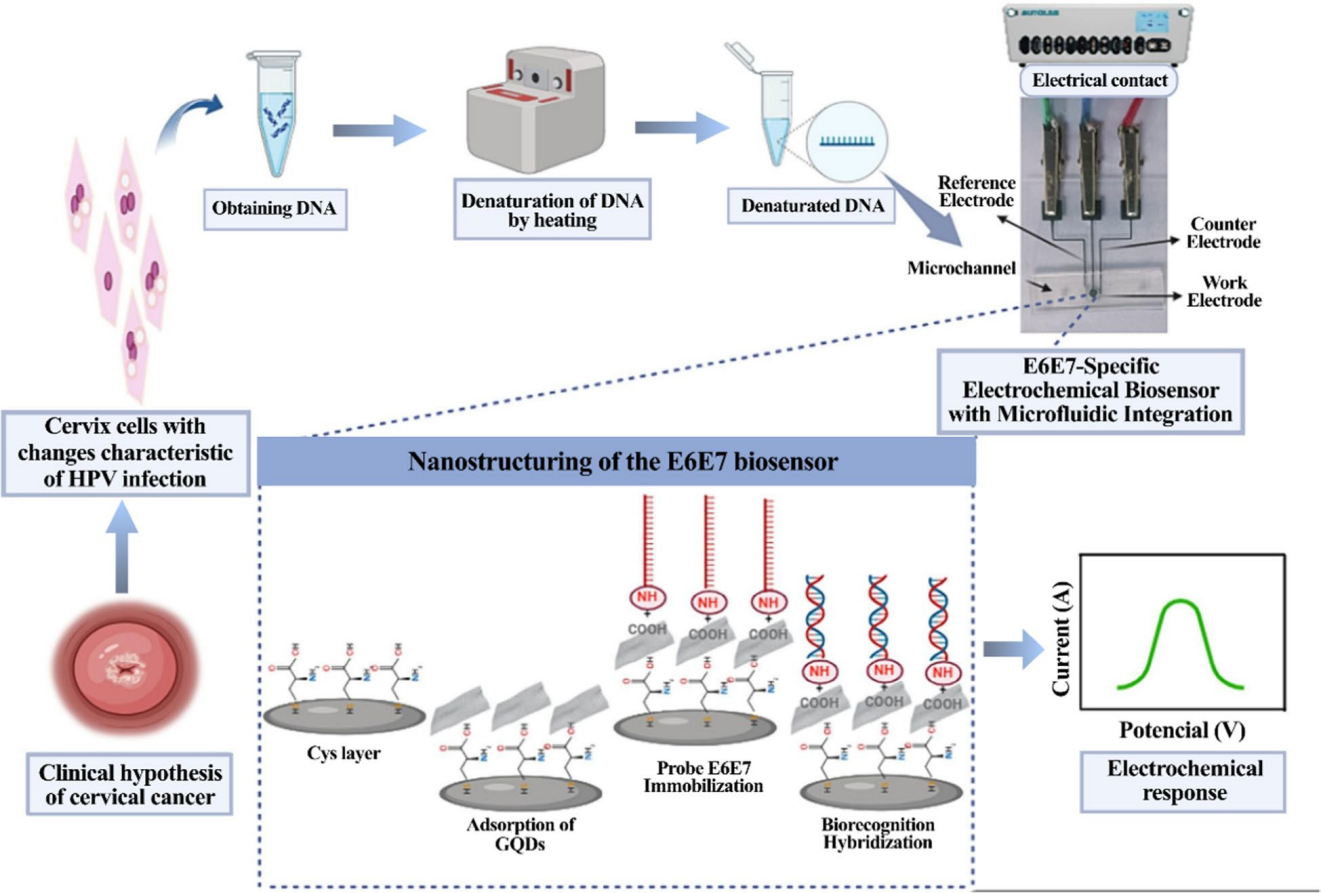
Detailed insight into the development and validation stages of the E6E7 microfluidic lab-on-chip genosensor. Reprinted with permission from [156]

The E6 and E7 oncogenes of human papillomavirus (HPV) drive carcinogenesis by disrupting cell cycle regulation, with E6 degrading p53 and E7 interfering with the Rb protein, leading to uncontrolled cell proliferation and cervical cancer. Advanced diagnostic methods are crucial for detecting HPV infections, differentiating genotypes, and identifying high-risk cases, enabling early intervention and targeted management to reduce HPV-related cancer incidence [154, 155]. Regarding this, Oliveira et al. introduced the design and validation of a label-free electrochemical genosensor integrated with a microfluidic system for the sensitive detection of E6 and E7 oncogenes in cervical smear samples. The construction process employed was predicated on a layer comprising cysteine (Cys) and GQDs, which are known to possess functional groups, augment surface area, and have notable electrochemical characteristics. Moreover, the Cys-GQDs matrix has played a remarkable role in immobilizing the DNA probe, thus creating a natural and biologically compatible setting. Biorecognition assays conducted on cervical scraping specimens have demonstrated a discernible variation in voltammetric response (Fig. 6). The biorecognition response for low-risk HPV strains was comparatively lower, with ΔI% values of 82.33% ± 0.29 for HPV06 and 80.65% ± 0.68 for HPV11 at a 1:100 dilution. In contrast, high-risk strains, HPV16 and HPV18, exhibited significantly higher ΔI% values of 96.65% ± 1.27 and 93% ± 0.026, respectively, at the same dilution. Following calibration via DPV analysis, the E6/E7 microfluidic LOC genosensor revealed an LOD of 26 fM and a quantification limit of 79.6 fM. Furthermore, the reproducibility of the E6E7 biosensor was evaluated by utilizing multiple biosensors on different days. Consequently, the biosensor exhibited notable reproducibility, with a 1.97% variation, and high repeatability, with a 0.455% variation. These findings underscore the biosensor’s robust bioanalytical performance. The biosensor’s ability to identify and quantify the E6 and E7 oncogenes is highly promising for clinical decision-making, offering rapid, and label-free detection with the potential for early intervention in HPV-related cervical lesions [156].

In a recent report, Dinani et al. presented a femtosecond laser conversion technique that was employed to fabricate a miniaturized LIG sensor array on a polyimide substrate and encapsulate it within the substrate. The compact device constituted a wearable microchip that facilitated the accurate surveillance of electrocardiography (ECG), electromyography, thermal readings, and glucose metrics within the spatial confines typically designated for a solitary sensor. The electrodes composed of femtosecond LIG (FSLIG) were altered to operate as sensors for glucose detection, and extensive electrochemical evaluations employing CV and CA techniques were conducted. To evaluate the performance of the FSLIG electrode as a glucose sensor, it was modified with GOx as a conventional approach. The results displayed remarkable electrochemical performance, attributed to its high porosity and doping process, with diffusion-controlled oxidation and reduction peaks (*R*2 > 0.998) and a linear relationship between peak current and glucose concentration (*R*2 = 0.99). Additionally, SEM imaging confirmed successful encapsulation, and interference tests with ascorbic and uric acid showed stable readings, proving the sensor’s reliability in biological environments. The glucose sensor, significantly smaller than prevailing commercial alternatives, exhibited superior durability, with merely a 3.86% signal variation after 100 cycles, thereby highlighting its stability and potential applicability in noninvasive, continuous health monitoring. The biocompatibility of the wearable biosensor was substantiated through a cytotoxicity assessment in accordance with ISO 10993–5 standards, during which L- 929 fibroblast cells exhibited no indications of toxicity or cellular lysis (reactivity grade 0), thereby affirming its suitability for incorporation into wearable health monitoring apparatuses. Nevertheless, future research should prioritize the advancement of multilayered printed circuit boards by integrating encapsulated graphene onto copper traces. This approach leverages graphem’s exceptional thermal conductivity and high-frequency performance to improve heat dissipation and signal integrity in innovative electronic devices. Encapsulation could also lead to the creation of compact, flexible sensors and sterile biomedical probes, with potential applications in aerospace and military sectors, opening new avenues for FSLIG-based technologies [157].

In another distinct study, Chen et al. harnessed electrochemical sensing chip technology for exceptionally sensitive detection of breast cancer exosomes. The electrochemical immunobiosensing microchip utilizes a one-step doping process of rGO with LIG to facilitate the quantitative assessment of exosomes. A laser engraver was used to create electrode patterns, followed by laser cutting of a polydimethylsiloxane layer to form sensing microcavities, which were then bonded to the electrodes. As the final construction step, polyethyleneimine (PEI)–rGO was deposited on the electrode, functionalized with glutaraldehyde and antibodies, and blocked with bovine serum albumin (BSA) to construct an electrochemical immunosensing chip. The electrochemical properties of LIG and rGO–LIG electrodes were analyzed using the CV method in a 0.1 M KCl solution containing 5 mM [Fe(CN)₆]3⁻/4⁻ at scan rates between 10 and 100 mV s⁻1. The redox peak currents increased with scan rate, showing a diffusion-controlled electron transfer process, and the electroactive surface areas, calculated using the Randles–Sevcik equation, were 0.182 cm2 for LIG and 0.271 cm2 for rGO–LIG. The results indicate that rGO doping significantly enhances the conductivity and electroactive surface area of the biosensing interface. Following the optimization of experimental conditions, the biosensor chip demonstrated a strong linear relationship between *R*_ct_ value and exosome concentration from 5 × 102 to 5 × 105 particles/μL, achieving a LOD of 166 particles/μL utilizing EIS analysis. The introduction of rGO explains the enhanced sensitivity by increasing the electrochemical surface area and antibody binding. Furthermore, the biochip underwent evaluation using clinical serum samples derived from 22 individuals diagnosed with breast cancer and 10 healthy subjects, revealing a markedly elevated exosome expression in the cancer-afflicted cohort (*P* < 0.0001). These findings underscore the biochip’s capability to proficiently differentiate between healthy individuals and those with breast cancer, thereby emphasizing its relevance for clinical diagnostic applications [158].

Both studies utilize GO to enhance the performance of electrochemical biosensors, though they target different applications and employ distinct fabrication methods. While both studies incorporate rGO to enhance sensor performance, Chen et al. focused on cancer biomarker detection using a microfluidic approach, whereas Dinani et al. developed a multi-sensor platform for comprehensive health monitoring. These applications underscore the versatility of GO-based materials in developing advanced biosensing technologies across diverse medical fields. In addition to these discussions, recent studies about graphene-based lab-on-a-chip platforms are tabulated in Table 2.

**Table 2:** Some selected studies on graphene-based lab-on-a-chip platforms and their applications

**Analyte**	**Electrode**	**Method**	**Linear Range**	**LOD**	**Application**	**REF**
Prostate-Specific Antigen	PSA-mAb/AuNFs/SPCE	EIC	0–100 ng/mL	0.28 ng/mL	Human serum	[153]
Urea	Urease/G-LOC	Potentiometry	1 − 50 mEq/L	0.2 mEq/L	Human serum Human saliva	[159]
K^+^	K^+^ ionophore/G-LOC		1 − 100 mEq/L	0.5 mEq/L		
Na^+^	Na^+^ ionophore/G-LOC		2 − 200 mEq/L	2.5 mEq/L		
E6/E7 Genes	Cys/GQDs/ssDNA E6E7/GCE	DPV	NS	26 fM	Plasmid and Cervical Scrapping	[156]
VP28	CE/AuNPs/activeSAM/BSA anti-VP28	EIS	0–60 ng/mL	2.38 ng/mL	Shrimp with WSSV	[160]
SARS-CoV- 2	ssDNA/PLA/Gr/3D-PPE	DPV	0.0001–0.5 µM	0.1 nM	Cerebrospinal fluid	[161]
Zearalenone	ZEN-Apt/GO/FTO	EIS	1–300 ng/mL	0.29 ng/mL	Corn	[162]
BRCA Exosomes	exo-cAb/rGO/LIG	EIS	5 × 10^2^–5 × 10^5^ particles/μL	166 particles/μL	BRCA patient serum	[158]
Phenylalanine	PheDH/ErGO/SPAuE	AMP	0–20 mg/dL	0.0524 mg/dL	Human plasma	[163]
Glucose	GOx/FSLIG	CA	5–40 mM	NS	Human skin	[157]
SARS-CoV- 2 spike S1 protein	Viral Antigens/rGO- 3DcC	EIS	10 fM – 30 nM	2.8 fM	Rabbit serum Fetal bovine serum	[164]
Gliadin	Gli4 Apt/MoS_2_/Gr/AuNPs/SPCE	DPV	4–250 nM	7 pM	Flour samples	[165]

Abbreviations: *3DcC*: 3D-printed COVID- 19 test chip, *3D-PPE*: 3D printing pencil electrode, *Apt*: Aptamer, BRCA: breast cancer, *ErGO*: electrochemically reduced graphene oxide, *Gli4*: gliadin, *G-LOC*: graphene-based LOC, *Gr*: graphene, *mAb*: monoclonal antibody, *MoS*_2_: molybdenum disulfide, *PheDH*: phenylalanine dehydrogenase, *PLA*: polylactic acid, *S1*: 5′-NH_2_-modified probe DNA, *SAM*: self-assembled monolayer, *SPAuE*: screen-printed gold electrodes, *VP28*: white spot syndrome virus envelope protein, *WSSV*: white spot syndrome virus, *ZEN*: zearalenone

### Point-of-care diagnostics

POC diagnostics refers to clinical testing and diagnostics performed at or near the point of care rather than in a centralized laboratory. The primary goal of POC diagnostics is to provide immediate results, enabling faster clinical decision-making and more efficient patient management. Enabling immediate findings for more rapid medical decision-making where efficient patient management is the primary goal of POC diagnostics [166]. Most common examples include ECG, which is portable and capable of monitoring heart activities. In a correlative manner, rapid diagnostic tests that have been widely used during the recent pandemic to diagnose COVID- 19 are among the most frequently utilized forms of mobile health technologies. The main advantage of POC testing is not only assuring faster clinical diagnosis but also establishing increased access and diminishing the costs of healthcare [167].

Electrochemical principles may also be engaged in such practical uses, offering rapid and on-the-spot results by utilizing their prominent features to track various biomarkers, metabolites, or pathogens in biospecimens by quantifying electrical signal outputs resulting from chemical reactions. At the core of electrochemical technologies in POC diagnostics, electrodes function as the sentinels of detection, responding to subtle shifts in electrical metrics, such as voltage, current, or impedance, when a target analyte interacts with the sensor’s outer responsive layer [168]. These sensors, like the finely tuned instruments of a symphony, are often composed of materials such as carbon, gold, or silver and are meticulously constructed with enzymes, antibodies, or nanoparticles to attain heightened specificity. Biosensors, a prominent class of electrochemical sensors, are tailored to recognize and analyze biochemical compounds, including carbohydrates, lipids, and nucleic acids (RNA or DNA), much like a magnet effortlessly drawing in its perfect counterpart [169]. Among these, the enzyme-based glucose meter stands as a hallmark of POC diagnostics, a tool of critical significance in managing diabetes mellitus, symbolizing the fusion of precision and accessibility in modern healthcare. Nevertheless, although many POC diagnostic instruments are precise, some may lack the reliability and capacity of laboratory assessments, requiring rigorous quality control assessments and regulatory approval to ensure consistent and effective outcomes outside traditional labs [170].

**Fig. 7:**
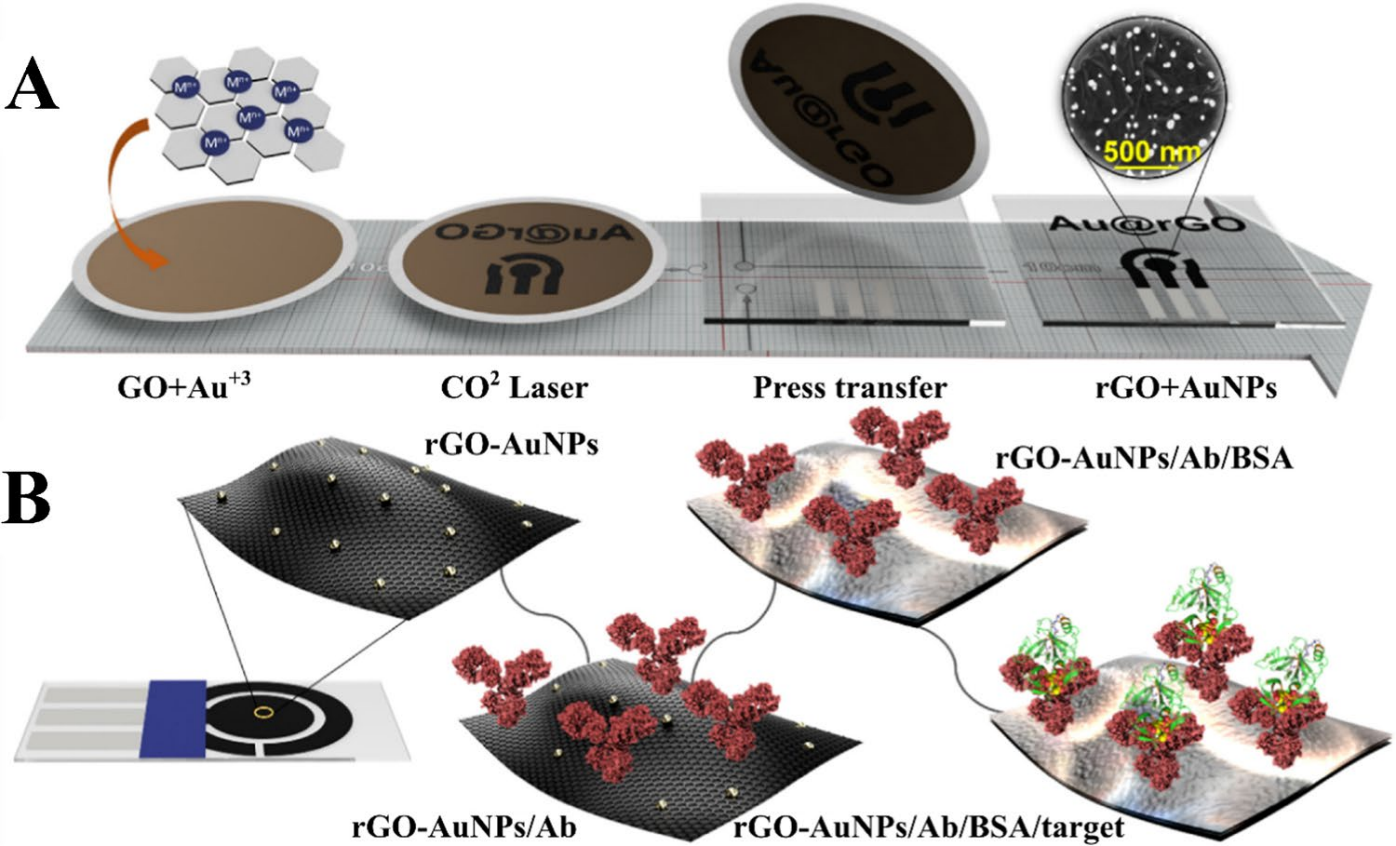
Schematic representation of the designed sensor. Reprinted with permission from [173]

Electrochemical nanobiosensors have emerged as pioneering tools in modern healthcare, offering a satisfactory solution for POC diagnostics. These advanced devices are capable of detecting specific biomarkers with enhanced sensitivity and precision in clinical settings, enclosed in a mobile and user-friendly design. Their key benefits include rapid response, simplicity, affordability, and feasibility for compact design, making them invaluable in clinical applications [171, 172]. Herein, laser-scribing technology stands as a promising approach, providing a simple, fast, and cost-efficient method for fabricating nanostructured electrodes in biosensing applications. By employing infrared (IR) lasers, experts managed to generate graphene-based nanostructured electrodes via laser stimulation of polymer underlayers like polysulfone, polyetherimide, and polyimide. This high-tech solution results in the production of graphene and graphene-metal NP nanocomposites. In a very recent study, Merkoçi’s research group proposed the rGO-AuNP electrodes modified by a laser-scribing method as the first capacitive immunosensor. The electrodes were meticulously immobilized with an anti-human IgG antibody, facilitating the identification of IgG antibodies in human serum and thereby validating the capacitance-based biosensing technique. Utilizing the altered biosensor signal, where capacitance shifts according to the desired amount, the group was able to develop an effective immunosensor rGO-AuNP electrodes to track the CA- 19–9 glycoprotein, which is a vital cancer biomarker. This demonstrates the functional usefulness of rGO-AuNP electrodes for recognizing CA- 19–9 glycoprotein (Fig. 7). The capacitance response diminished as the concentration of CA- 19–9 glycoprotein increased, exhibiting a direct correlation applicable for analytical investigations, with a linearity range of 0 to 300 U/mL. The determined LOD was 8.9 U/mL, with the quantification threshold established at 29.6 U/mL, calculated using the 3-sigma and 10-sigma standards, respectively. These thresholds are critical in medicinal diagnostics since a CA- 19–9 glycoprotein level lower than 37 U/mL is considered to be standard in healthy individuals, whereas equal or higher levels are indicative of a potential cancer association. The analytical findings obtained by the electrochemical capacitance spectroscopy (ECS) method demonstrated high inter-electrode reproducibility (RSD = 2.67%) and stability, with minimal variations in relative response after blank sample exposure (RSD = 0.60%). Additionally, the intra-sensor repeatability and inter-sensor repeatability for CA- 19–9 recognition were excellent, with RSDs of 0.3% and 5.8%, respectively. While long-term stability was not assessed, freshly prepared biosensors were used to ensure optimal bioreceptor integrity and functionality. These analytical features indicate that this novel capacitive nanoscale biosensor, utilizing rGO-AuNPs, shows promise for diagnostic application in the detection of CA- 19–9 glycoprotein, operating with no labels or added reagents [173].

Leveraging their enhanced sensitivity and selectivity, Rafati et al. utilized microRNAs for the early identification of colorectal cancer biomarkers, specifically targeting microRNA- 223 (miR- 223). As an innovative label-free nanostructured electrochemical biosensor, GQDs are decorated with AuNPs (Au-GQD-NS), which was followed by a consecutive immobilization of thiolated DNA probes (Cap- 223). For the characterization studies, FESEM, EDAX, AFM, and electrochemical methods were engaged to analyze the nanostructured surface further. Electrochemical techniques, such as DPV, EIS, and CV, were particularly employed to assess the electroactivity of the substrate, the immobilization of Cap- 223 on Au-GQD-NPs, and the subsequent hybridization with miR- 223. Consequently, a broad array of microRNA- 223 concentrations ranging from zeptomolar to micromolar was assessed, yielding an ultra-low LOD of 0.024 attomolar for the nanostructured biosensor. To evaluate the sensitivity and performance of the immunosensor, DPV and EIS measurements were performed with miR- 223 concentrations from 10 zM to 200 µM. The data revealed a linear correlation between *R*_ct_ and the logarithm of miR- 223 concentration, demonstrating the sensor’s ability to detect miR- 223 at low traces. The probe was tested for miR- 223 detection using a human-extracted serum with a 2% serum dilution in PBS, minimizing interference and optimizing sensitivity. Results showed a low RSD percentage, indicating the biosensor’s reliability for detecting miR- 223 in real human samples, with the potential for early colorectal cancer diagnosis [174]. Rafati et al. targeted miR- 223 for early colorectal cancer diagnosis, employing DNA probe hybridization, while Merkoçi’s group focused on detecting CA- 19–9 glycoprotein using antibody-antigen interactions. Both studies leverage the synergistic effects of AuNPs with rGO and GQDs, respectively, to enhance biosensor performance. The rGO-AuNP integration focuses on capacitive immunosensing, utilizing antibody-antigen interactions to detect glycoproteins, which are crucial in cancer diagnostics. In contrast, the Au-GQD-NP integration emphasizes electrochemical DNA hybridization, targeting specific microRNAs associated with colorectal cancer. AuNPs are frequently integrated with nanomaterials like rGO and GQDs to enhance the performance of electrochemical biosensors. These combinations exploit the unique properties of each component, resulting in biosensors with improved sensitivity, selectivity, and stability.

Acute myocardial infarction (AMI) is a life-threatening condition that causes irreversible damage to myocardial cells, potentially leading to heart failure. Therefore, early and accurate diagnosis is critical to reduce mortality and improve patient outcomes. While cardiac troponin T (cTnT) is a widely used biomarker for AMI, its specificity is constrained, as elevated concentrations might also be observed in ischemic heart disease. Furthermore, the delayed response of protein-based biomarkers like cTnT does not meet the urgent diagnostic needs of AMI. Ongoing research points to microRNAs (miRNAs), particularly miRNA- 208a, as promising early molecular biological markers. miRNA- 208a is exclusively specific to cardiac tissue and is measurable within the plasma of AMI patients in less than 1 h of onset, offering resilient myocardial particularity, prompt detection, and better stability compared to traditional biomarkers. This positions miRNA- 208a as a valuable candidate for advancing the swiftness and accuracy of AMI diagnosis [175, 176]. The pursuit of ultra-sensitive biomarker recognition is a central pillar in global biomedicine studies, acting as the driving force for processes in early clinical diagnostics. Expanding semiconductor technology has positioned field-effect transistor (FET) sensors utilizing 2D nanomaterials like graphene as leading candidates for ultra-sensitive micro-nano sensors. Graphene’s atomic thickness, steady chemical attributes, vast surface expanse, and ease of surface coating render it ideal for engineering-sensitive interaction points. Although graphene-FETs (GFETs) are a breakthrough, the body of structured research is scarce on designing ultra-sensitive GFETs for biological sensing and optimizing device structures for high sensitivity and low noise. Herein, Hu et al. engineered a revolutionary GFET biosensor, meticulously refining detection parameters and crafting a state-of-the-art portable system designed for unparalleled ultra-sensitive, rapid, and highly specific detection of the early AMI biomarker miRNA- 208a. The CVP method was implemented to synthesize graphene. The transmittance analysis indicates that the graphene film maintains consistent light absorption across the 400 to 780 nm range, revealing a transmittance of approximately 97% at 550 nm. This value aligns closely with the transmittance reported for monolayer graphene in existing studies, confirming the monolayer nature of the CVD-grown graphene. An ssDNA sequence was selected as a complementary probe. This approach enabled a detection range of 0.01–1 pM for miR208a, with a LOD of 5.3 fM in 40 min. As a portable device, the sensor demonstrated superior performance compared to its alternatives, showing a concentration range of 0.1–100 pM. A potentiometric assay was conducted to determine selectivity coefficients, suggesting that graphene exhibits non-targeted adsorption to miRNA- 208a. To summarize, this work offers a robust method for swift and ultrasensitive quantification of miR208a, with promising applications for monitoring other diagnostic biomarkers. GFET biosensors are anticipated to facilitate nucleic acid detection without the need for amplification, paving the way for compact and portable diagnostic systems, which are ideal for POC applications [177]. In addition to these discussions, recent studies about graphene-based POC testing platforms are tabulated in Table 3.

**Table 3:** Some selected studies on graphene-based point of care testing platforms and their applications

**Analyte**	**Electrode**	**Method**	**Linear Range**	**LOD**	**Application**	**REF **
*Cryptosporidium parvum*	ssDNA Apts/3D Au-NMIs	DPV	10 − 100.000 oocysts/mL	5 oocysts/mL	Tap water Stool media	[178]
*Mycobacterium tuberculosis*	Anti-MPT64 Abs/1,5-DAN/Glut/GFET	AMP	0.001–10 pg/mL	1 fg/mL	Diluted serum	[179]
Procalcitonin	Anti-PCT Ab/rGO-AuNP-PEDOT:PSS@CFP	CA	1 × 10^3^–6 × 10^7^ fg/mL	0.28 × 10^3^ fg/mL	NS	[180]
miRNA- 223	Cap- 223/AuNPs-GQD-FTO	DPV EIS	10 zM–200 nM	0.024 aM	Human serum	[174]
Hydrocortisone	ALP/Anti-Cortisol Ab/Au–IrO_2_NFs/rGO/SPE	DPV	11.3– 140.4 ng/mL	8.9 ng/mL	NS	[181]
Glucose	GOx/LIG	CA	Up to 1.5 mM	13.7 μM	Synthetic sweat	[182]
Lactate	LOx/LIG	CA	10 μM–5 mM	28 μM		
K^+^	ISE/LIG	Potentiometry	Up to 1 M	10^–4.5^ M		
Electrolytes	PB/chitosan	EIS	1 mM–1 M	10^–3.5^ M		
S-protein RBD	VHH-E Nb/Pyrene-E-TP/LSGE	EIS	150 pM–15 nM	7.68 pM	NS	[183]
MCF- 7 Cell Exosomes	Anti-CD- 9 Ab/AuNPs@rGO/NiNf/exoPAD	DPV	500–1 × 10^7^ Exosome/µL	110 Exosome/µL	Human serum	[184]
Tetracycline	Apt-SGGT	EIS	NS	2.073 pM	Milk	[185]
				100 pM		
Vitamin D	Anti-Vit-D Ab/Ti_3_C_2_T_x_Mxene/LIG	DPV	0.1–500 ng/mL	1 pg/mL	Human serum	[186]
*E. coli*	ssDNA Apt/ DES/GO/AuNPs	FET	3–3 × 10^6^ CFU/mL	3 CFU/mL	Human serum	[187]

Abbreviations: *ALP*: alkaline phosphatase, *Au–IrO2 NFs*: Au–IrO2 nanoflowers, *CD- 9 Ab*: α-human CD9 antibody, *DES*: deep eutectic solvents, *E. coli*: Escherichia coli, *E-TP*: E-Tag peptide, *exoPAD*: exosome-sensing paper-based analytical device, *Glut*: glutaraldehyde, *ISE*: ion-selective electrodes, *Nb*: nanobody, *NF*: nickel foam, *NiNf*: nickel nanofoam, *NMIs*: nano/micro-islands, *PAD*: paper-based analytical device, *PCT*: procalcitonin, *PEDOT*: poly(3,4- ethylenedioxythiophene), *PSS*: polystyrene sulfonate, *SGGT*: solution-gated graphene transistors, *S-protein RBD*: spike protein receptor-binding domain, *VHH*: variable domain of the heavy chain of a heavy-chain-only antibody, *Vit-D*: vitamin D

### Implantable devices

Implantable medical devices are designed to be inserted permanently or temporarily into the body to monitor, treat, or support diverse biological functions. They are a fundamental part of modern healthcare, providing instantaneous data and direct therapeutic interventions for chronic conditions, disabilities, and life-threatening illnesses. There exists a wide array of implantable devices, each designed for particular body systems and tailored for various diagnostic and therapeutic applications [188]. These devices are distinguished by their ability to serve different functions across cardiovascular, neurological, orthopedic, and metabolic systems, enhancing patient care through targeted, continuous monitoring or intervention. Examples include cardiac devices (e.g., pacemakers, implantable cardioverter-defibrillators), pharmaceutical delivery platforms such as insulin pumps, and electrochemical sensors to track specific biomarkers. Electrochemical detection involves the use of sensing devices that exploit electrochemical reactions, namely redox, to quantitatively assess the amount of targeted analytes in a sample, offering high sensitivity and specificity for operations in diagnostic evaluations and environmental monitoring [189].

More importantly, implantable sensors are constructed by biocompatible materials to minimize possible immune responses or rejection by the body. In order to ensure optimal long-term functionality and stability in the physiological environment, it is essential to select appropriate materials, which may include platinum, gold, and specific polymers [190]. Recent advancements in nanotechnology and microfabrication have eased the way for the development of implantable electrochemical sensors that are exceptionally smaller, more efficient, and less invasive. This process of miniaturization not only enhances sensor performance but also minimizes patient discomfort, facilitating the deployment of these technologies in otherwise hard-to-access areas within the body. However, achieving long-term stability and ensuring a reliable, enduring power supply for these sensors remains a significant challenge [191].

Beyond the aforementioned applications, microneedles represent a cutting-edge technology that integrates microneedle arrays with electrochemical sensors, achieving minimally invasive and exceptionally sensitive real-time monitoring of biochemical markers by accessing interstitial fluid, blood, or other biological specimens through painless penetration of the skin’s outer layer without causing significant discomfort [192]. Furthermore, the detection and measurement of inorganic compounds, such as heavy metals, transition metals, and electrolytes, in bodily fluids and environmental sources are vital for maintaining both public and individual health. Hence, it is inevitable to advance novel sensing technologies with different arrays of modifications due to the increased demand for sensitive, selective, and accessible tools in on-site and laboratory practices [193].

Leveraging these technologies, Xie et al. proposed dual-mode implantable microelectrode arrays (IMEAs) to track the synergistic link between the hippocampus and cortex of epileptic rats. The study incorporated carboxylated graphene oxide to facilitate the directional coupling of glutamate oxidase (GluOx) biomolecules to neural microelectrodes, thereby enabling the real-time monitoring of glutamate concentration and electrophysiology in the cortex and hippocampus of epileptic rats under ruthenium-bipyridine-triphenylphosphine caged-gamma aminobutyric acid (RuBi-GABA) modulation. The strategically engineered PtNPs/rGO-GluOx with 1,3-phenylenediamine (mPD) interface facilitated highly sensitive glutamate detection at a low operating potential of 0.1 V. This configuration achieved a sensitivity of 141.00 ± 5.66 nA μM⁻^1^ mm⁻^2^ and a LOD of 0.3 μM, effectively addressing common challenges associated with enzyme immobilization on microelectrodes. Specifically, it mitigated issues such as surface heterogeneity, high operational voltages, and suboptimal sensitivity, thereby enhancing overall sensor performance. In addition to electrochemical performance tests, in vivo experiments were also conducted utilizing pilocarpine-induced epileptic rat models for real-time neural and glutamate detection. After implanting electrodes and optical fibers, low-dose pilocarpine (3 mg/kg) induced seizures, causing abnormal neural signals and increased glutamate levels. To suppress seizures, RuBi-GABA (5 mM, 10 μL) was injected into the hippocampus and activated with blue light, while the IMEA recorded real-time neural and glutamate changes. The study revealed that glutamate levels rose before electrophysiological signals in the pre-seizure phase, indicating that glutamate-GABA imbalance is a key factor in epilepsy, with excessive glutamate triggering widespread neuronal excitation. The paper also concluded that glutamate accumulation was more pronounced in the hippocampus than in the cortex, supporting its role in epilepsy. The preaccumulation of glutamate before abnormal neural bursts was also more evident in the hippocampus, suggesting that glutamate changes in this region could serve as biomarkers for early epilepsy detection [194].

In a subsequent investigative study, Wu et al. sought to further elucidate the enigmatic nature of the nervous system through an implantable aptamer-functionalized graphene microtransistor probe (~ 390 μm wide, ~ 76.8 μm thick) for real-time neurochemical monitoring, particularly dopamine detection. The probe consists of four graphene microtransistors (50 μm × 50 μm each), providing high spatial resolution and minimal tissue damage compared to traditional microdialysis probes. Aptamers, known for their high selectivity and stability, were functionalized onto graphene via π − π stacking to enable specific dopamine detection while overcoming the Debye screening limitation. The probe’s soft mechanics and lightweight design (31 mg) allowed for deep brain implantation in small animals with reduced tissue inflammation and glial scar formation. The dopamine-sensitive microtransistors demonstrated a rightward shift in the Dirac point upon surface functionalization, thereby confirming the effective binding of aptamers and the capacity for real-time dopamine monitoring. With a fixed gate voltage of 50 mV, the sensor’s current increased with dopamine concentration, showing a detection limit of 10 pM and a dynamic range of 10 pM to 100 μM. The sensor’s response followed a Langmuir adsorption isotherm, with a Kd of 6.19 nM, indicating a high aptamer affinity for dopamine. More notably, the probe exhibited superior sensitivity, with an analytical range spanning 10 pM, and achieved remarkable molecular specificity, exhibiting over 19-fold selectivity for dopamine relative to norepinephrine. Additionally, it displayed spatiotemporal precision, with cellular-scale resolution, an on-time of 2.09 s, and an off-time of 5.38 s, which all empower real-time monitoring of dopamine fluctuations in vivo. In mouse models, the probe effectively monitored dopamine release induced by pharmacological stimulation, providing valuable insights into the neurochemical processes underlying behavior and neural activity. Nevertheless, future advancements should focus on enhancing resistance to biofouling, enabling precise monitoring of dopamine release in the nucleus accumbens, and confirming dopamine release through methods such as fast-scan cyclic voltammetry or fluorescent sensors [195].

### Other applications

Since graphene-based electrochemical biosensing strategies could not be narrowed down to POC diagnostics, LOC platforms, wearables, or implantable technologies, this section will discuss the other innovative applications that were reported within the last 5 years.

As an example, in a recent investigation conducted by Patil et al., amine-functionalized magnetic-rGO (MgrGO-NH_2_) composite material was engaged utilizing a sandwich-type immunosensor for the identification of the breast cancer bioindicator, carbohydrate antigen 15–3 (CA 15–3). Timely detection is essential in breast cancer, as it is with all cancer types, for improving outcomes and enabling more effective treatment strategies. Biomarkers are promising elements for tracking cancer cells and facilitating timely diagnosis, which is followed by cost-effective therapy strategies, minimally invasive treatments, and, more importantly, improved survival rates [196, 197]. Development of the new immunosensor was focused on the modification of a platinum SPE not only with MgrGO-NH_2_ composite but also with amine-functionalized nanocomposite of Fe_3_O_4_ NPs and multiwalled carbon nanotubes (Fe_3_O_4_-MWCNTs-NH_2_) as well as CA 15–3 antibodies with 1% BSA solution to obtain sandwich-configurated electrochemical immunosensor. Transmission electron microscopy images, selected area electron diffraction patterns, XPS survey spectrums, and Raman spectrums successfully validated the structural and morphological properties of the ultimate sensor. DPV technique confirmed the electrochemical performance of the newly engineered immunosensor, revealing a LOD value of 0.0001 U/mL and a relatively wide concentration range of 0.0005 to 100 U/mL. Following the approved protocols, human serum specimens were acquired from patients with breast cancer to evaluate the electrochemical functionality of the novel immunosensor in real-world clinical applications. Besides, the developed immunosensor demonstrated successful identification of CA 15–3 in real serum samples, as evidenced by its favorable comparison to enzyme-linked immunosorbent assay results, low RSDs ranging from 1.19 to 2.84%, and high recovery rates between 97.3% and 101.7% at a 95% statistical confidence threshold. Thus, both methodologies demonstrated superior and promising performance in their respective detection of biomarkers with efficient detection limits and rapid analysis for early diagnosis of the above-mentioned cancer varieties [198, 199].

*Acinetobacter baumannii (A. baumannii)*, akin to a surreptitious stealthy architect, evades the immune system by resisting disinfectants and constructing biofilm fortresses on a variety of physiological surfaces. Its ability to hide within these protective communities has led different organizations, such as the WHO and FDA, to identify it as a critical objective for new antimicrobial strategies, especially as its multidrug resistance, including to carbapenems, has surged over the past two decades. The COVID- 19 health crisis has further amplified the threat of *A. baumannii* infections in emergency care units, emphasizing the urgency for robust diagnostic methods beyond current labor-intensive and resource-heavy techniques. Electrochemical biosensors, renowned for their compactness and exceptional sensitivity, function like microscopic detectives uncovering the electrical whispers of electrochemical processes. These involve Faradaic reactions, where currents shift during redox processes, and non-Faradaic processes, such as EIS, where impedance arises from the dance of charges in the double-layer capacitance. Functioning with the precision of a Swiss watchmaker assembling intricate gears, these sensors are not only capable of identifying but also quantifying objective molecules effectively [200, 201]. Singhal et al. fabricated SPCEs with both GO and rGO as the immobilization platform to address the aforementioned challenges. Moreover, an aptamer targeting bacteria was adopted as the biorecognition element, which selectively interacts with the target. This coupling results in a reduction at the peak response of the redox system, as the biomolecules associated with the electrode surface restrict the electron transfer of the redox agent. More interestingly, this newly engineered label-free rGO aptasensor was compatible with POC diagnostics revealing an excellent sensitivity with a remarkable LOD (10 CFU/mL). The resultant increase in sensitivity might be ascribed to multiple critical factors, such as the structural anomalies generated during reduction, the enlarged outer area, and the enhanced electron transfer kinetics. Moreover, stability tests proved the outstanding resilience of *A. baumannii*/Apt/®GO/SPCE sensor by preserving its performance after 4 weeks of storage. As a result of the experiments with both GO and rGO fabricated electrodes, it was concluded that rGO-coated electrodes displayed greater sensitivity with lower detection limits compared with GO modification. The superior performance was ascribed to the remarkable characteristics of rGO, such as its faster electron transfer kinetics, expanded surface area, structural imperfections, and π–π stacking interactions. Accordingly, these factors enriched the binding characteristics of the aptamers, leading to a more effective detection capability for *A. baumannii.* Exquisitely adjusting the sensor to recognize a wider array of pathogens may prove conducive to a more comprehensive outcome. Thereby, research will be able to cast a broader net in combating infectious diseases, ultimately elevating public health and maintaining public safety [202]. In summary, while both studies leverage the unique properties of graphene oxide derivatives to enhance biosensor functionality, Patil et al. focus on an immunosensor configuration using functionalized magnetic-rGO composites for cancer biomarker detection, whereas Singhal et al. emphasize the optimization of electrode modification demonstrating that rGO outperforms GO in pathogen detection applications. In addition to these discussions, recent studies about graphene-based electrochemical biosensing platforms are tabulated in Table 4.

**Table 4:** A selection of studies regarding graphene-based electrochemical biosensors and their applications

Analyte	Electrode	Method	Linear range	LOD	Application	REF
Ag^+^	MB-CRO/ErGO-GCE	DPV	1 fM–100 nM	0.83 fM	Pond and tap water	[203]
Glutamate	GLDH/rGO/NiF	DPV	5–300 µM	0.1 µM	NS	[204]
Cortisol	al-Au/GO-COOH/Cor-MIP/GCE	DPV	1 × 10^−3^–1 × 10^−14^ M	0.61 × 10^−14^ M	Human serum	[205]
Matrix Metalloproteinase 2	Anti-MMP2 Apts/ErGO-IDE	EIS	10 pg/mL–10 ng/mL	3.32 pg/mL	Human serum	[206]
LRG1	pyrene-rGO/LRG1-specific peptides/AuDE	SWV	100 pg/mL–100 ng/mL	75 pg/mL	Human serum	[207]
3-hydroxydecanoic acid	GCE/rGO/MnNPs/L-Ser@MIP	DPV	1.0 × 10^−6^–50 × 10^−6^ μg/mL	4.2 × 10^−7^ μg/mL	Guava agro-waste	[208]
*A. baumannii*	AB/Apt/rGO/SPCE	EIS	10–10^9^ CFU/mL	10 CFU/mL	NS	[202]
	AB/Apt/GO/SPCE	DPV				
		CV				
Erythropoietin	EPO-Ab/CuO/N-rGO/GCE	DPV	10^−2^–10^4^ ng/L	3 pg/L	Human serum	[209]
Prostate-specific antigen	PSA-Apts/rGO/g-C_3_N_4_/AuNPs/GCE	SWV	2.5–12.5 pM	0.44 fM	Human serum	[210]
Glucose	GOx/PdONPs-rGO/C-PE	DPV	0.005–13.75 mM	0.046 μM	Collage and professional athletes, tomato	[211]
*Helicobacter pylori*	rGO/Au/PTP NPs-Apt/GCE	EIS	10–900 nM	0.0080 μM	Human serum	[212]
		SWV		0.0067 μM		
miRNA- 21	ssDNA/GO/Gr/FTO	DPV	10 fM ~ 1 nM	3.18 fM	NS	[213]
CA- 19–9 glycoprotein	rGO-AuNP/IgGAb/BSA/SPE	ECS	0–300 U/mL	8.9 U/mL	Human IgG antibodies	[173]
Monocrotophos	GO/Ab/FTO	DPV	1 ppt–1 ppm	0.49 ppm	Vegetable extract Pond water	[214]
CEA	AuNP/AuND/CS-rGO/Anti-CEA/GCE	DPV	0.0001–10 ng/mL	0.001 pg/mL	Human serum	[215]
MicroRNA	GO-Fe_3_O_4_/PEDOT/DNA/GCE	CC	10^−15^–10^−6^ M	5.18 × 10^−15^ M	Human serum	[216]
		AMP		7.36 × 10^−15^ M		
*Mycoplasma pneumoniae*	GO/Cu–MOF/PY/EDC-NHS/anti-*M.p.*&*L.p*	DPV	1 pg/mL– 100 ng/mL	9.4 pg/mL	Tap water	[217]
*Legionella pneumophila*				8.3 pg/mL		
17α-ethinylestradiol	Au/p(nBA-co-NAS)/Apt/SPE	DPV	1 × 10^−12^ M–1 × 10^−6^ M	1.7 × 10^−13^ M	Lake water	[218]
Lactate	Au/rGO/PtNPs/CS@ LOx/Nf/MNs	CA	0–10,000 μM	2.04 μM	Artificial ISF Human serum	[219]
Pb^+2^	MB-AuNPs-ssDNA/Apt/ PAMAM@Au/GO/SPCE	DPV	0.5 pM–500 nM	5 fM	Lead ion samples	[220]
IL- 6	anti-IL- 6 Ab/CS/POP-AQ/GO/SPCE	SWV	0.005–5 ng/mL	0.0040 ng/mL	Human serum	[221]
D-dimer	anti-D-dimer Ab/CS/POP-AQ/GO/SPCE		2.5–100 ng/mL	0.844 ng/mL		
Ferritin	anti-ferritin Ab/CS/POP-AQ/GO/SPCE		2.5–100 ng/mL	0.806 ng/mL		
CA 15–3	MgrGO-NH_2_/Ab_1_/ BSA/Ag/Fe_3_O_4_-MWCNTs-NH_2_-Ab_2_/SPE	DPV	0.0005–100 U/mL	0.0001 U/mL	BCP human serum	[199]
Thrombin	TBLP/TA-PEI-GO/AuE	DPV	NS	0.48 nM	Human serum and plasma Mouse serum and plasma	[222]
	TBCP/TA-PEI-GO/AuE			0.61 nM		
Carbendazim	ROMP/AquaMet/Ab/CBZ/BSA/Apt GO-PEI/GCE	SWV	1 pg/mL–100 ng/mL	7.80 fg/mL	Food samples	[223]
Cold-resistant tRNA	RNA/BSA/ssRNA/Au@MoS_2_/Co(OH)_2_/GCE	DPV	0.001 nM–100 µM	0.18 pM	Winter wheat	[224]
Anti-*Leishmania* antibodies	*Leishmania infantum/*AuNPs/rGO/SPCE	CV	NS	5.58 mg/mL	Symptomatic and asymptomatic patients	[225]
Uric acid	UOx/AuNPs–GO–CS cry/PB-PEDOT:PSS/SPCE	AMP	5.0–300 µM	1.88 µM	Human plasma	[226]
*E. coli*-ssDNA	S1-CS/dCuO@rGO/DNA/GCE	DPV	0.001–37.5 pM	3.89 × 10^–1^ fM	NS	[227]
Hg^+2^	MCH/cDNA-MgGO-AuNPs/MGCE	SWV	5 pM–100 nM	3.14 pM	River water Lake water	[228]
Dopamine	Apt/AuNPs/PEDOT-ErGO/GCE	DPV	5.0–200 μM	1.0 μM	Fetal bovine serum	[229]
Cancer antigen 125	Ab/CuCo-ONSs@AuNPs/GCE	DPV	1 × 10^−7^ U/mL–1 × 10^−3^ U/mL	3.9 × 10^–8^ U/mL	Human serum	[230]
Glucose	GOx/N-GQD/PrGE	CA	1 mM–9 mM	0.098 mM	Human serum	[231]
Hsp16.3	cAb/GrCOOH/AuPs/PAD	SWV	0.01–30 ng/mL	0.03 ng/mL	Human serum	[232]
SKBR3 cell line	rGO/Fe_3_O_4_/Nf/PANI Herceptin Ab/GCE	SWV	10^2^–10^6^ cells/mL	5 cells/mL	Cell lines	[233]
Guanine	TiO_2_/poly(_L_-lysine)/GrQDs/GCE	SWV	1.0–35.0 µM	0.56 µM	Milk Urine	[234]
Adenine				0.81 µM		
Alpha-fetoprotein	AFP-Ab/LIG/Gr-PANI	DPV	4–400 ng/mL	1.15 ng/mL	Human serum	[235]
17β-estradiol	E2-Ab/LIG/Gr-PANI		20–400 pg/mL	0.96 pg/mL		
Acrylamide	HbNPs/GQDs/PGE	CV	10–120 nM	2.70 nM	Potato crisps	[236]
Hg^2+^	Apt/AuNPs/PLL/BP-PG/GCE	DPV	1–10.000 nM	0.045 nM	River water Soil Vegetables	[237]
miR208a	ETA/ssDNA/PBASE/Gr/GFET	AMP	0.01–1 pM	5.3 fM	NS	[177]

Abbreviations: *1,5-DAN*: 1,5-diaminopahthalene, *AB*: *Acinetobacter baumannii*, *AFP*: alpha-fetoprotein, *al-Au*: allylated gold nanoparticle, *AQ*: asymmetry quotients, *AuDE*: gold disk electrodes, *AuND*: gold nanodendrites, *AuPs*: gold particles, *BP-PG*: black phosphorus-porous graphene, *CBZ*: carbendazim, *CC*: chronocoulometry, *cDNA*: capture DNA, *CE/AuNPs*: AuNP-s deposited chip, *CEA*: carcinoembryonic antigen, *CFP*: cellulose fiber paper, *ChOx*: cholesterol oxidase, *Co(OH)*_2_: cobalt hydroxide nanosheets, *CoO NWs*: CoO nanowires, *C-PE*: cellulose substrate, *cry*: cCryogel, *CuCo-ONSs*: copper-cobalt oxide nanosheets, *CuO*: copper oxide, *d-CuO*: *HKUST- 1-*derived -CuO, *E2*: 17β-estradiol, *EDC*: 1-ethyl- 3-(3-dimethylaminopropyl) carbodiimide hydrochloride, *EPO-Ab*: anti-rhu, erythropoietin antibody, *g-C*_3_*N*_4_: graphitic carbon nitride, *GLDH*: glutamate dehydrogenase, *GO-COOH*: carboxylated graphene oxide, *GrCOOH*: carboxyl graphene, *GrQDs*: graphene core–shell quantum dots, *HbNPs*: hemoglobin nanoparticles, *Hsp16.3*: heat shock protein, *IDE*: gold interdigitated electrodes, *L.p.*: Legionella pneumophila, *LRG1*: leucine-rich alpha- 2 glycoprotein- 1, *L-Ser*: poly-LL-serine, *M.p.*: *Mycoplasma pneumoniae*, *MB*: methylene blue, *MCH*: 6-Mercapto- 1-hexanol, *MGCE*: magnetic glassy carbon electrode, *MIP*: molecularly imprinted polymer, *MMP2*: matrix metalloproteinase 2, *MnNPs*: manganese nanoparticles, *MNs*: microneedles, *Nf*: nafion, *NHS*: N-hydroxy succinimide, *NiF*: nickel foam, *N-rGO*: nitrogen-modified reduced graphene oxide, *PAMAM*: polyamide-amine, *PANI*: polyaniline, *PBASE*: 1-pyrenebutanoic acid succinimidyl ester, *PdONPs*: palladium(II) oxide nanoparticles, *PGE*: pencil graphite electrode, *PLL*: polylysine, *POP*: porous organic polymer, *PrGE*: printed graphene electrodes, *PTP*: polythiophene, *ROMP*: ring-opening metathesis polymerization, *ssRNA*: single-stranded RNA, *TBCP*: thrombin-binding cyclic peptide, *TBLP*: thrombin-binding linear peptide, *TiO2*: titanium dioxide, *tRNA*: target RNA, *UOx*: uricase,

## Current insights, challenges, and limitations

Analyzing the molecular and vibrational properties of 2D materials presents significant challenges due to their extremely small sample sizes. However, various techniques have been developed to support their identification and characterization. This section presents a structured approach for studying monolayer-thick materials and highlights emerging yet underutilized methods that combine structural and spectroscopic data at a localized level. One of the main difficulties in studying monolayer materials is their preparation and detection. The most straightforward way to obtain 2D layers from van der Waals solids, such as graphene, metal chalcogenides, and germanium hydride, is through mechanical exfoliation. This process involves peeling thin sheets from bulk crystals and transferring them onto different substrates [238]. The resulting flakes are typically around 10 μm in size and can vary in thickness from a single monolayer to multiple layers. Optical microscopy is a widely used and efficient method for identifying these flakes. In most cases, dielectric-coated silicon dioxide/silicon (SiO₂/Si) substrates are employed for visualization. The interference effect between the dielectric layers enhances color contrast, making it easier to distinguish monolayer structures from multilayer ones. To achieve the highest contrast, the dielectric coating must be carefully adjusted, ideally within 5 nm of the optimal thickness. However, since the refractive indices of many new materials are not well established, experimental determination of the best thickness is often necessary. This is usually done by exfoliating the material onto substrates with different dielectric layer thicknesses [8].

2D materials offer several advantages over conventional bulk materials, such as silicon, particularly in the development of scalable electronic devices. Their superior electrostatic properties and reduced thickness allow for optimized electrostatic screening and an improved quantum capacitance threshold. In modern silicon-on-insulator transistors, reducing the channel length requires thinning the semiconductor layer to maintain effective electrostatic control [53]. However, key differences between bulk and monolayer materials are still under investigation, especially in areas such as many-body interactions, phonon transport, interfacial electron coupling, excitonic effects, defect behavior, and substrate interactions. At the monolayer level, environmental factors can significantly influence experimental outcomes, making it challenging to isolate the intrinsic properties of these materials. Improving techniques for growing high-quality, large-area monolayers could enhance our fundamental understanding and lead to new discoveries. Recent research on 2D materials has contributed to emerging fields such as spintronics and valleytronics, which have the potential to enable innovative technological applications. Despite their promise, graphene-based materials raise concerns regarding their biocompatibility, which must be addressed before they can be widely used in clinical settings. Compared to CNTs, which have been extensively studied for biomedical applications like biosensors, drug delivery, cancer treatment, and medical imaging, graphene presents both benefits and challenges. While GO can be produced more affordably, its functionalization with polymers such as polyethylene glycol (PEG) remains costly. Additionally, unlike CNTs, which have strong resonance Raman scattering and near-infrared (NIR) photoluminescence useful for medical imaging, graphene lacks these natural optical properties and requires external labeling for effective imaging. However, some studies suggest that reduced rGO exhibits greater photothermal responsiveness than CNTs, despite its lower NIR absorption [239].

Biocompatibility studies suggest that unmodified graphene oxide GO tends to accumulate in the lungs after intravenous administration, leading to dose-dependent pulmonary toxicity. However, modifying its surface, such as through PEGylation, significantly improves its biocompatibility by altering its biodistribution and reducing toxicity. PEG-functionalized nanographene oxide (NGO-PEG) is more stable in physiological conditions, is primarily cleared through the reticuloendothelial system, and does not accumulate in the lungs, showing minimal long-term toxicity [240]. Despite these findings, further research is needed to fully understand the long-term biological behavior and toxicological impact of graphene derivatives. Graphene’s interaction with biological systems is largely influenced by its physicochemical properties, including size, surface charge, particulate state, layer number, and functional groups. Once introduced into a biological environment, graphene rapidly adsorbs biomolecules, forming a protein corona that alters its physicochemical characteristics and affects biological interactions. This process influences cellular uptake, immune responses, and potential cytotoxic effects. Some in vitro studies have shown that graphene exposure can induce oxidative stress and cytotoxicity in human cell lines in a dose-dependent manner, but the exact mechanisms behind graphene-induced toxicity remain unclear. Therefore, further systematic research is needed. As graphene continues to be explored for biomedical applications, comprehensive in vitro and in vivo toxicological studies will be essential to ensuring its safe use in clinical settings [241].

## Conclusions and future perspectives

From the past to the present, after this long journey, the recent extension of the human lifespan has been accompanied by an increase in diseases within modern society. Alongside this rise, the diagnosis and treatment of these diseases, as well as the monitoring of environmental pollutants that lead to life-threatening illnesses, have marked the dawn of a new era of advancement through the convergence of novel technologies and disciplines. Determining physiological and biochemical processes has become critical in diagnosing, identifying, and treating these diseases. Disease diagnostics often rely on bodily fluids such as blood, saliva, sweat, and urine, where biomarkers such as hormones, antibodies, and proteins exhibit variations in levels and types depending on the illness. In addition to these, serum glucose, triglycerides, and glycated hemoglobin are recognized as early indicators of disease. In recent years, biomarkers have been instrumental in the early diagnosis of several conditions, including cancer, diabetes, AIDS, infectious diseases, cardiovascular diseases, stroke, and neurodegenerative disorders. They have also been essential in researching drug responses and evaluating treatment efficacy. Early detection is crucial as it can significantly reduce treatment costs in the advanced stages of illness. Until now, various methods, including conventional spectroscopic techniques and chromatographic methods, have been developed for the detection of biomolecules, drugs, or heavy metals. However, these approaches often encounter significant interference issues. Consequently, there is a critical need for more sensitive, portable, low-cost, simple, and selective sensors based on nanocomposites, particularly from the perspective of health and safety.

Graphene offers numerous remarkable benefits; however, it is essential to acknowledge that its technology readiness level (TRL) remains relatively low across most application domains. To increase TRLs and advance the development of more sophisticated prototype systems for commercial use, further research is necessary. In particular, to accelerate the industrialization of 2D materials and maximize their commercial potential, complementary technologies such as artificial intelligence and blockchain must be developed and integrated with these devices. As a result, TRLs are expected to rise significantly in the near future [242]. Graphene-based electrochemical biosensors, which have demonstrated promising performance under controlled laboratory conditions (TRL 3), will require additional modifications, such as filtration, pre-dilution, and temperature correction, to be successfully translated into real-world field applications. According to the Graphene Flagship Annual Report, several graphene-enabled sensor platforms are progressing toward TRL 5–6, having achieved important proof-of-concept and preclinical validation. Despite these advancements, challenges remain, particularly in scalable manufacturing, long-term stability testing, and obtaining the necessary regulatory approvals before these biosensors can achieve full commercial deployment (TRL 9) [242].

Following the discovery of graphene, the graphene family has expanded continually, revealing new variants with distinct structures and properties, each contributing to the development of carbon-based materials. In the development of electrochemical nanosensors, nanomaterials such as graphene/graphene oxide, carbon, and carbon nitride nanotubes are utilized to enhance sensitivity, providing an advanced and robust platform for addressing these challenges. In this regard, highly selective, specific, and reliable sensors have been developed. Moreover, the sensors obtained must be rapid, sensitive, portable, and cost-effective. Continuous biomedical monitoring systems and non-invasive tracking technologies, including point-of-care devices that allow patients or their caregivers to perform tests, have improved significantly over the past two decades. Electrochemical methods, known for their portability, selectivity, and remarkable sensitivity, provide both patients and professionals with considerable convenience in point-of-care diagnostics. A diverse array of nanomaterials, such as nanoparticles, quantum dots, fullerenes, and graphene, are being utilized in research for this purpose. These materials are deposited onto electrode surfaces to increase surface area and catalytic activity, thereby improving detection limits. Thus, there is an urgent demand for more sensitive, portable, cost-effective, simple, and selective sensors based on nanocomposites, particularly in terms of health and safety. Electrochemical sensing is highly sensitive to electroactive molecules, offering detection selectivity as different molecules oxidize or reduce at varying potentials, thus enhancing adaptability, which is a defining feature of electrical detection. In conclusion, this review is a comprehensive review of the historical evolution of the graphene narrative and its underlying scientific principles.

The widespread utilization of graphene-based materials in clinical biosensors highlights their exceptional physicochemical properties, which enhance sensor performance in terms of electrochemical conductivity, surface area, and biocompatibility. A notable trend observed in biosensor design is the frequent combination of these nanomaterials with gold-based nanostructures, such as AuNPs, AuE, and gold-modified substrates. This integration is primarily driven by gold’s excellent biocompatibility, chemical stability, and strong affinity for thiol and amine groups, which enhance biomolecule immobilization and stability. This dramatic enhancement not only significantly lowers the detection limits, sometimes reaching the femtomolar range, but also accelerates sensor response times. Such improvements are critical for developing ultra-sensitive clinical biosensors that can detect trace amounts of biomarkers, thereby enabling earlier diagnosis and more effective disease monitoring. This synergistic effect is a key reason behind the widespread integration of rGO, GO, and MWCNTs with gold in next-generation biosensing platforms. The diverse range of biosensor architectures incorporating these materials suggests their adaptability for detecting various clinical biomarkers, including proteins (e.g., interleukins, ferritin, and D-dimer), nucleic acids (e.g., RNA, ssDNA, and aptamers), and small molecules (e.g., glucose and uric acid).

Despite these advantages, challenges remain in ensuring the reproducibility, long-term stability, and clinical validation of biosensors utilizing these nanomaterials. Variability in synthesis methods, surface functionalization, and batch-to-batch consistency can impact sensor performance, necessitating standardized fabrication protocols. Furthermore, potential toxicity and biocompatibility concerns, particularly for MWCNTs and graphene derivatives, must be thoroughly investigated before clinical translation. Overall, the predominant use of graphene-based biosensors underscores their critical role in advancing clinical biosensors. Future research should focus on optimizing material synthesis, improving functionalization strategies, and conducting rigorous biocompatibility assessments to facilitate their widespread adoption in biomedical diagnostics.

## Data Availability

No datasets were generated or analysed during the current study.
